# Supplementary material for: Cannabidiol as Self-Assembly Inducer for Anticancer Drug-Based Nanoparticles
Source: Molecules. 2022 Dec 23;28(1):112. doi: 10.3390/molecules28010112 (PMC9822096; doi:10.3390/molecules28010112)
Supplement: Supplementary file 1 [file molecules-28-00112-s001.zip › molecules-2056547-supplementary.pdf]

Supplementary Information

Cannabidiol as Self-Assembly Inducer for the Obtainment of Anticancer Drug Based Nanoparticles

Eleonora Colombo [1,2,†], Davide Andrea Coppini [1,3,†], Laura Polito [4], Umberto Ciriello [5], Giuseppe Paladino [5], Mariafrancesca Hyeraci [6], Maria Luisa Di Paolo [7], Giulia Nordio [6,8], Lisa Dalla Via [6,8,*] and Daniele Passarella [1,*]

1 Dipartimento di Chimica, Università degli Studi di Milano, Via Golgi 19, 20133 Milano, Italy
2 Ann Romney Center for Neurologic Diseases, Department of Neurology, Brigham and Women's Hospital and Harvard Medical School, Boston, MA 02115, USA
3 Instituto de Productos Naturales y Agrobiología (IPNA), CSIC, 38206 La Laguna, Tenerife, Spain
4 Istituto di Scienze e Tecnologie Chimiche (SCITEC) "Giulio Natta", Consiglio Nazionale delle Ricerche (CNR), Via G. Fantoli 16/15, I-20138 Milan, Italy
5 LINNEA SA, 6595 Riazzino (TI), Switzerland
6 Dipartimento di Scienze del Farmaco, Università degli Studi di Padova, via F. Marzolo 5, 35131 Padova, Italy
7 Dipartimento di Medicina Molecolare, Università degli Studi di Padova, via G. Colombo 3, 35131 Padova, Italy
8 Consorzio Interuniversitario Nazionale per la Scienza e la Tecnologia dei Materiali, via G. Giusti 9, 50121 Firenze, Italy
* Correspondence: daniele.passarella@unimi.it; lisa.dallavia@unipd.it
† These authors contributed equally to this work.

^{1}H-NMR and ^{13}C NMR spectra

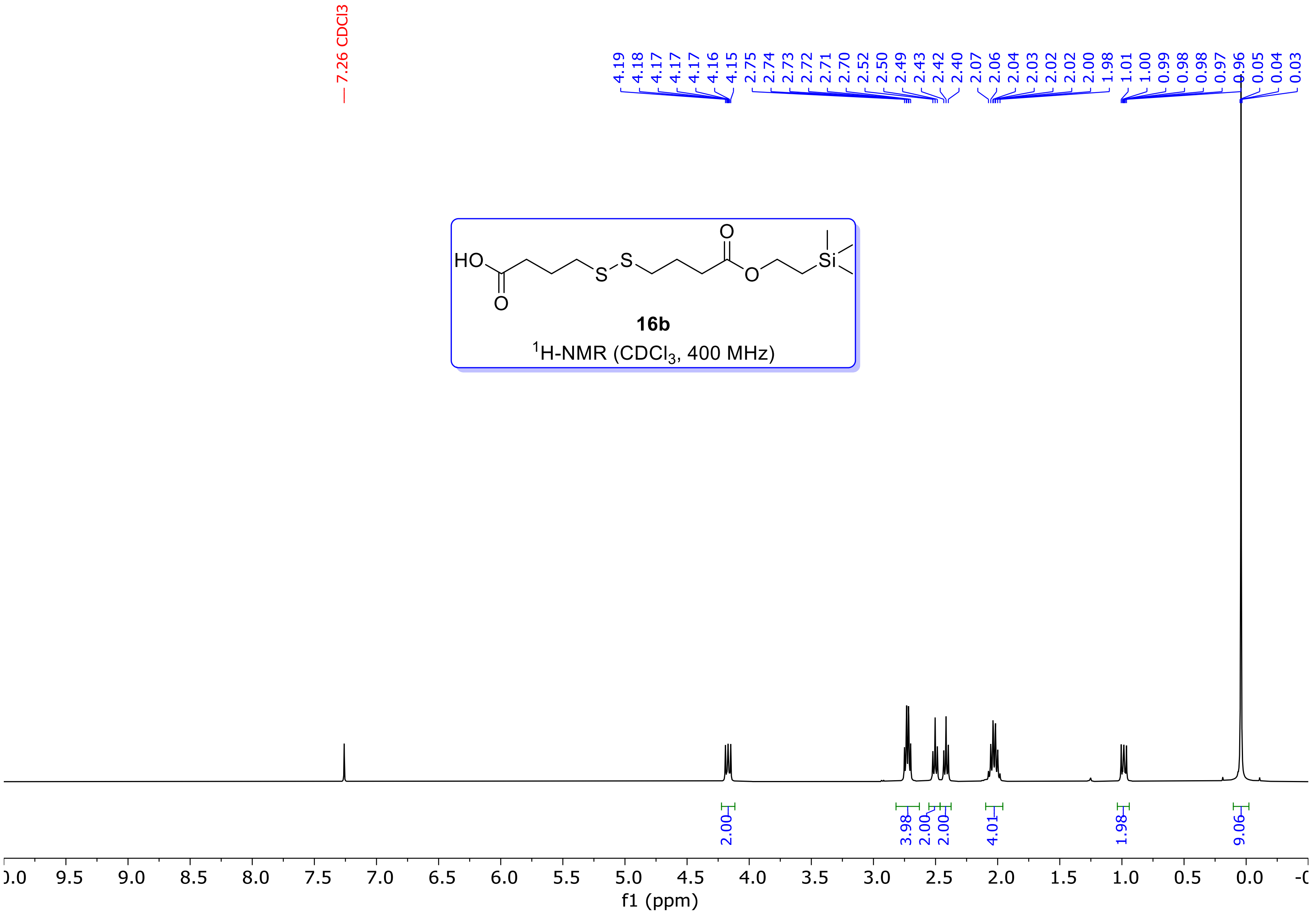

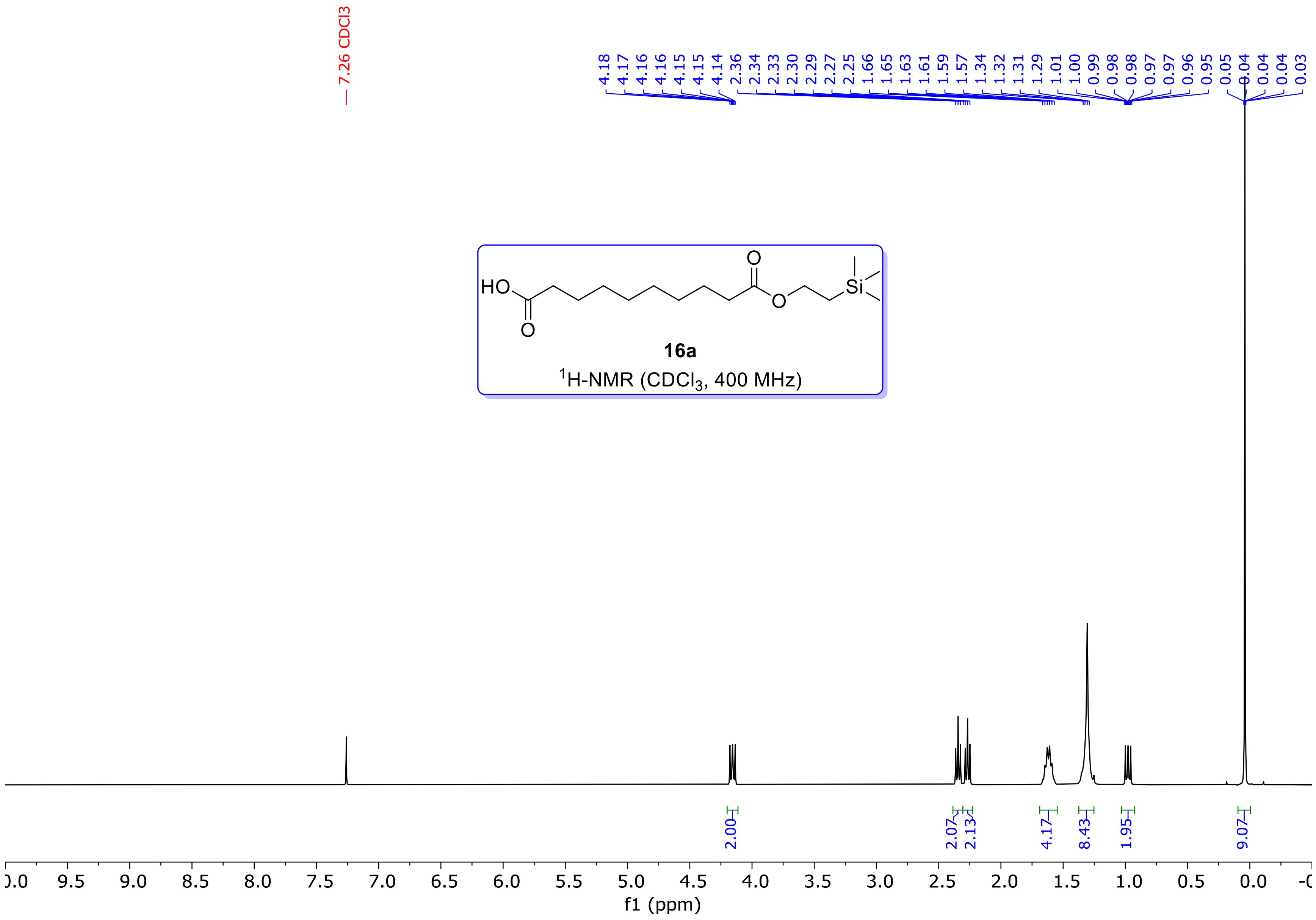
7.26 CDCl3
4.18 4.17 4.16 4.16 4.15 4.15 4.14 2.36 2.34 2.33 2.30 2.29 2.27 2.25 1.66 1.65 1.63 1.61 1.59 1.57 1.34 1.32 1.31 1.29 1.01 1.00 0.99 0.98 0.98 0.97 0.97 0.96 0.95 0.05 0.04 0.04 0.04 0.03
HO
O
O
O
Si
16a
1H-NMR (CDCl3, 400 MHz)
2.00
2.07
2.13
4.17
8.43
1.95
9.07
9.5 9.0 8.5 8.0 7.5 7.0 6.5 6.0 5.5 5.0 4.5 4.0 3.5 3.0 2.5 2.0 1.5 1.0 0.5 0.0
f1 (ppm)

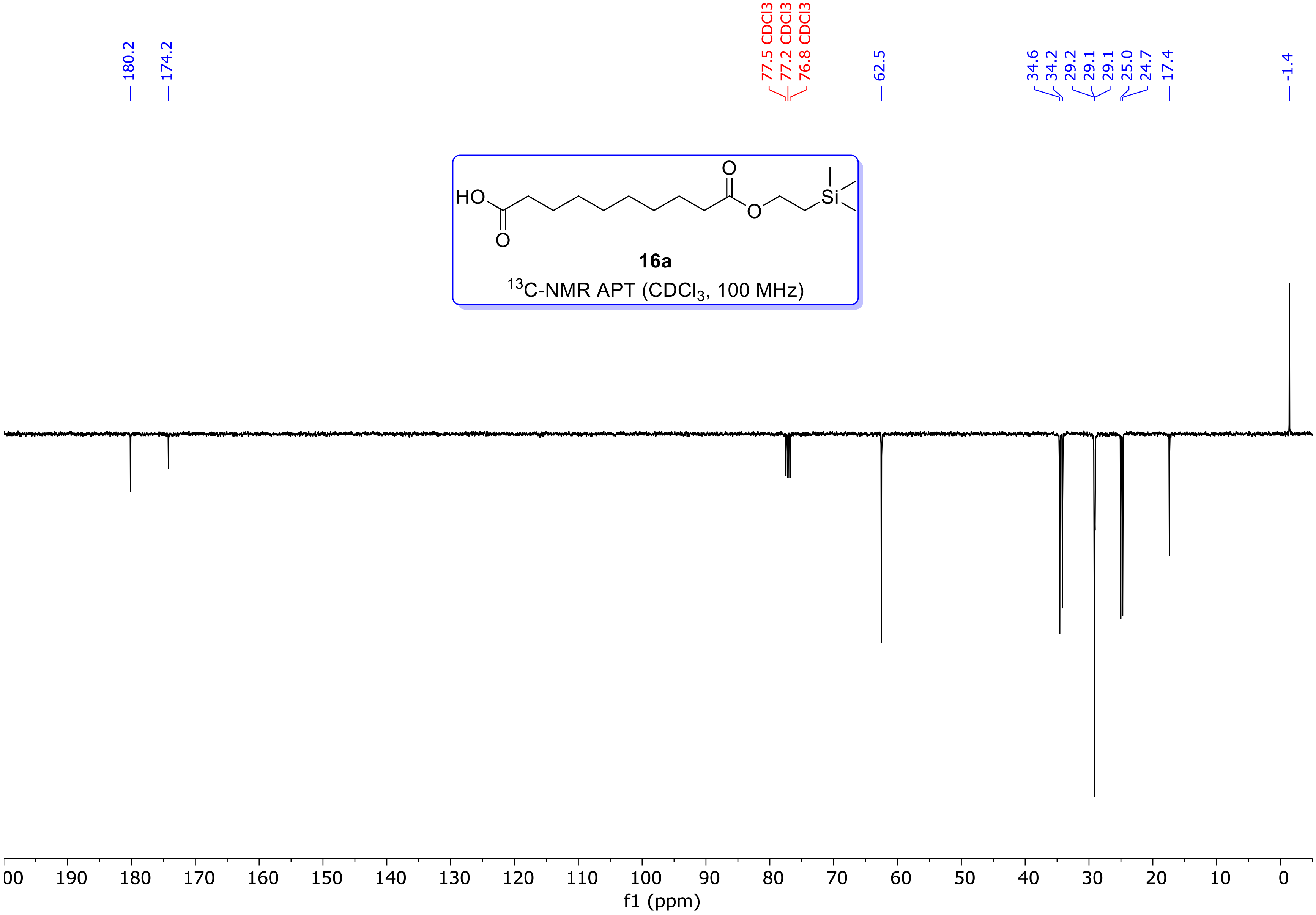
180.2
174.2
77.5 CDCl3
77.2 CDCl3
76.8 CDCl3
62.5
34.6
34.2
29.2
29.1
29.1
25.0
24.7
17.4
-1.4
HO
O
O
O
Si
16a
13C-NMR APT (CDCl3, 100 MHz)
00
190
180
170
160
150
140
130
120
110
100
90
80
70
60
50
40
30
20
10
0
f1 (ppm)

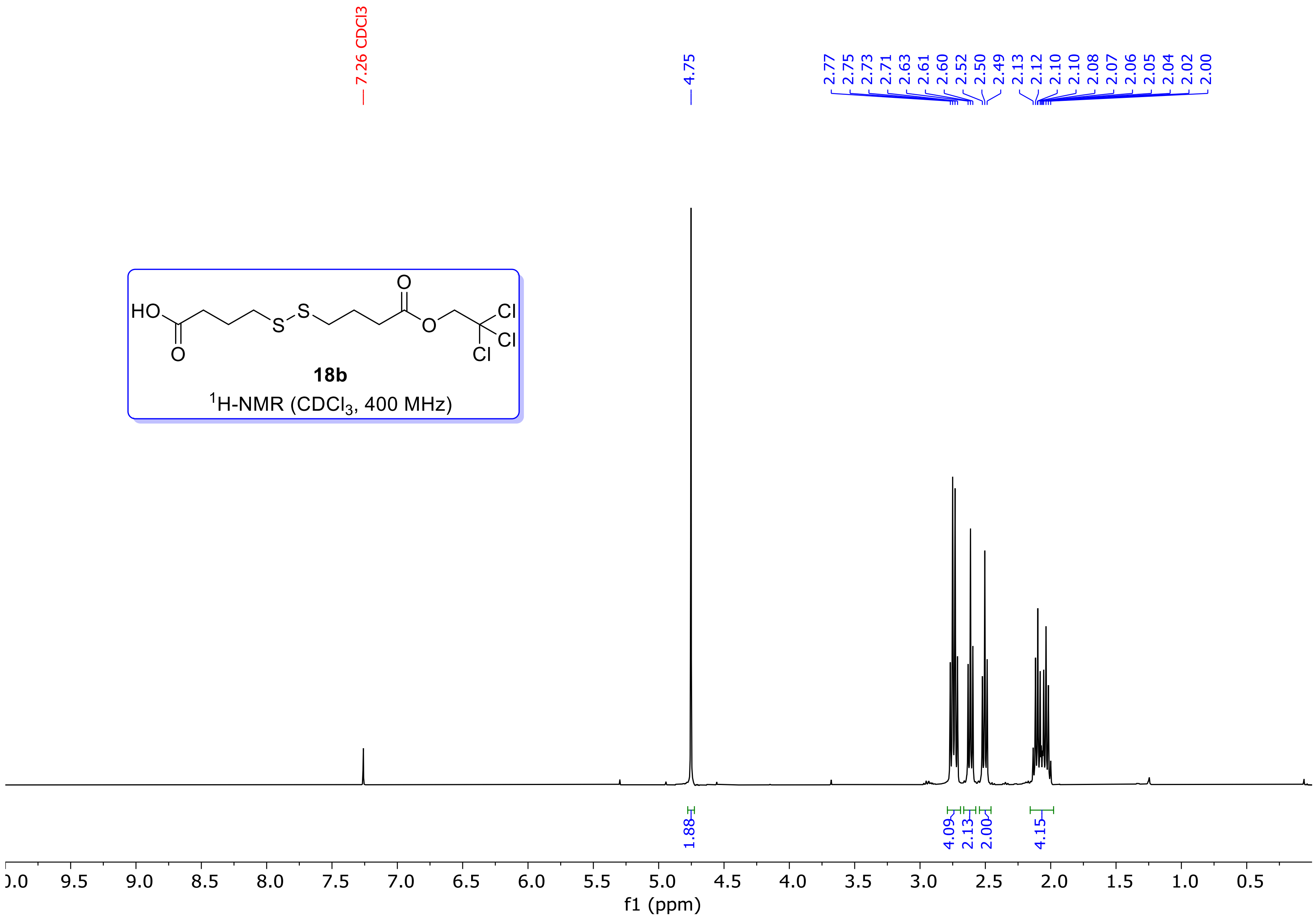
7.26 CDCl3
4.75
2.77 2.75 2.73 2.71 2.63 2.61 2.60 2.52 2.50 2.49 2.13 2.12 2.10 2.10 2.08 2.07 2.06 2.05 2.04 2.02 2.00
HO
S
S
O
O
O
Cl
Cl
Cl
18b
1H-NMR (CDCl3, 400 MHz)
1.88
4.09
2.13
2.00
4.15
10.0 9.5 9.0 8.5 8.0 7.5 7.0 6.5 6.0 5.5 5.0 4.5 4.0 3.5 3.0 2.5 2.0 1.5 1.0 0.5
f1 (ppm)

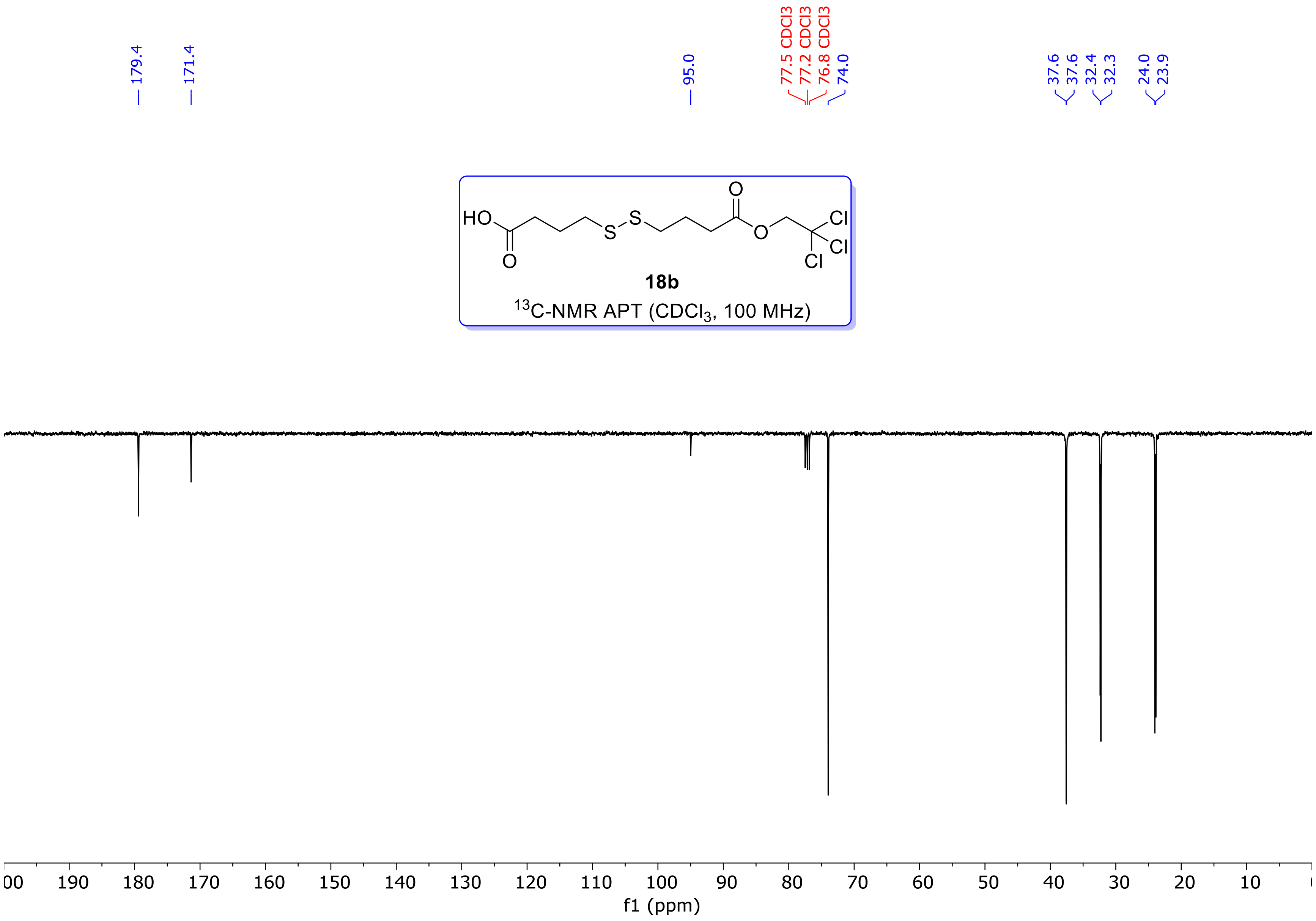

179.4
171.4
95.0
77.5 CDCl3
77.2 CDCl3
76.8 CDCl3
74.0
37.6
37.6
32.4
32.3
24.0
23.9
HO
O
S
S
O
O
Cl
Cl
Cl
18b
13C-NMR APT (CDCl3, 100 MHz)
190
180
170
160
150
140
130
120
110
100
90
80
70
60
50
40
30
20
10
f1 (ppm)

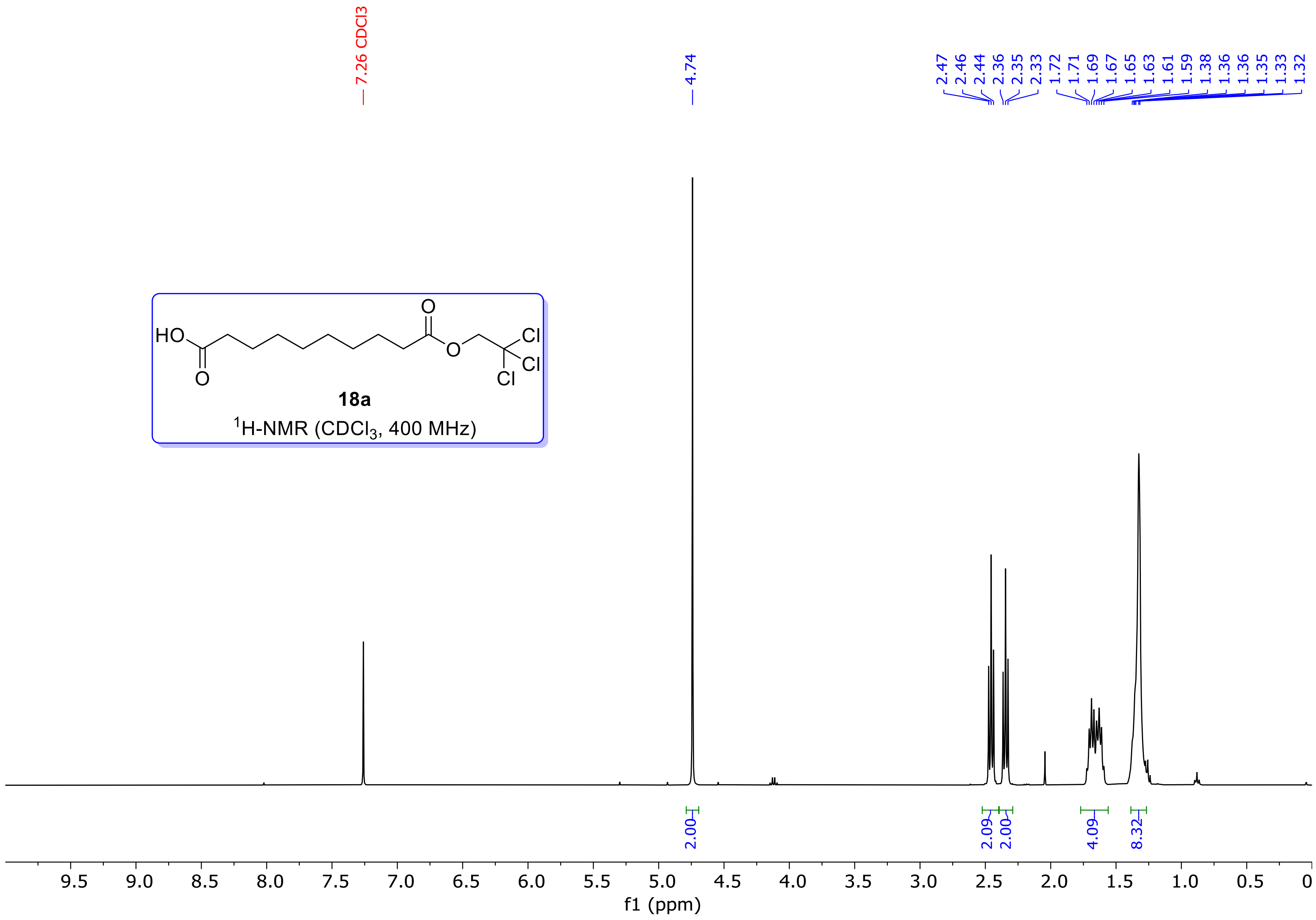
7.26 CDCl3
4.74
2.47
2.46
2.44
2.36
2.35
2.33
1.72
1.71
1.69
1.67
1.65
1.63
1.61
1.59
1.38
1.36
1.36
1.35
1.33
1.32
HO
O
O
O
Cl
Cl
Cl
18a
¹H-NMR (CDCl₃, 400 MHz)
2.00
2.09
2.00
4.09
8.32
9.5
9.0
8.5
8.0
7.5
7.0
6.5
6.0
5.5
5.0
4.5
4.0
3.5
3.0
2.5
2.0
1.5
1.0
0.5
0
f1 (ppm)

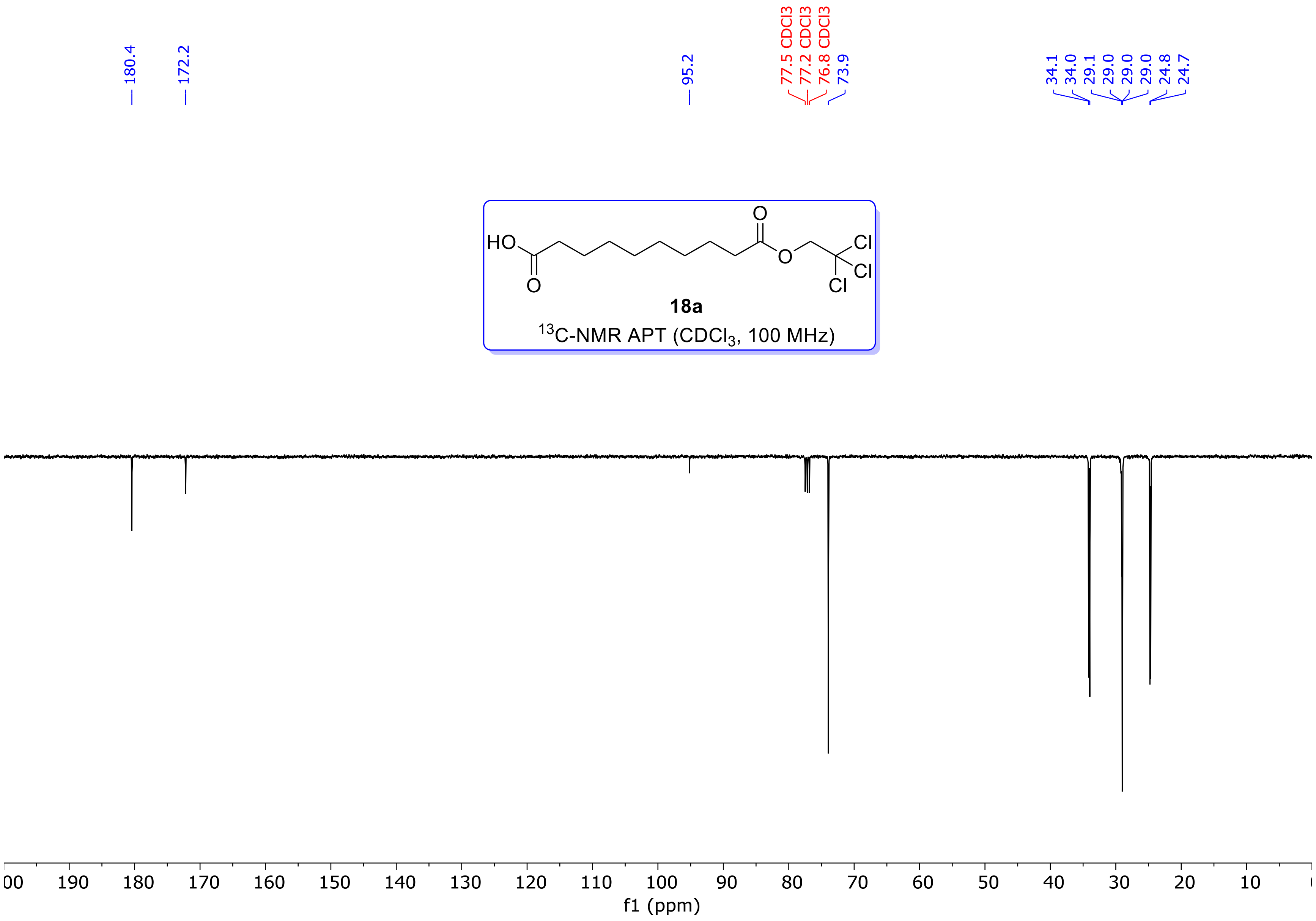
180.4
172.2
95.2
77.5 CDCl3
77.2 CDCl3
76.8 CDCl3
73.9
34.1
34.0
29.1
29.0
29.0
29.0
24.8
24.7
HO
O
O
O
Cl
Cl
Cl
18a
13C-NMR APT (CDCl3, 100 MHz)
f1 (ppm)

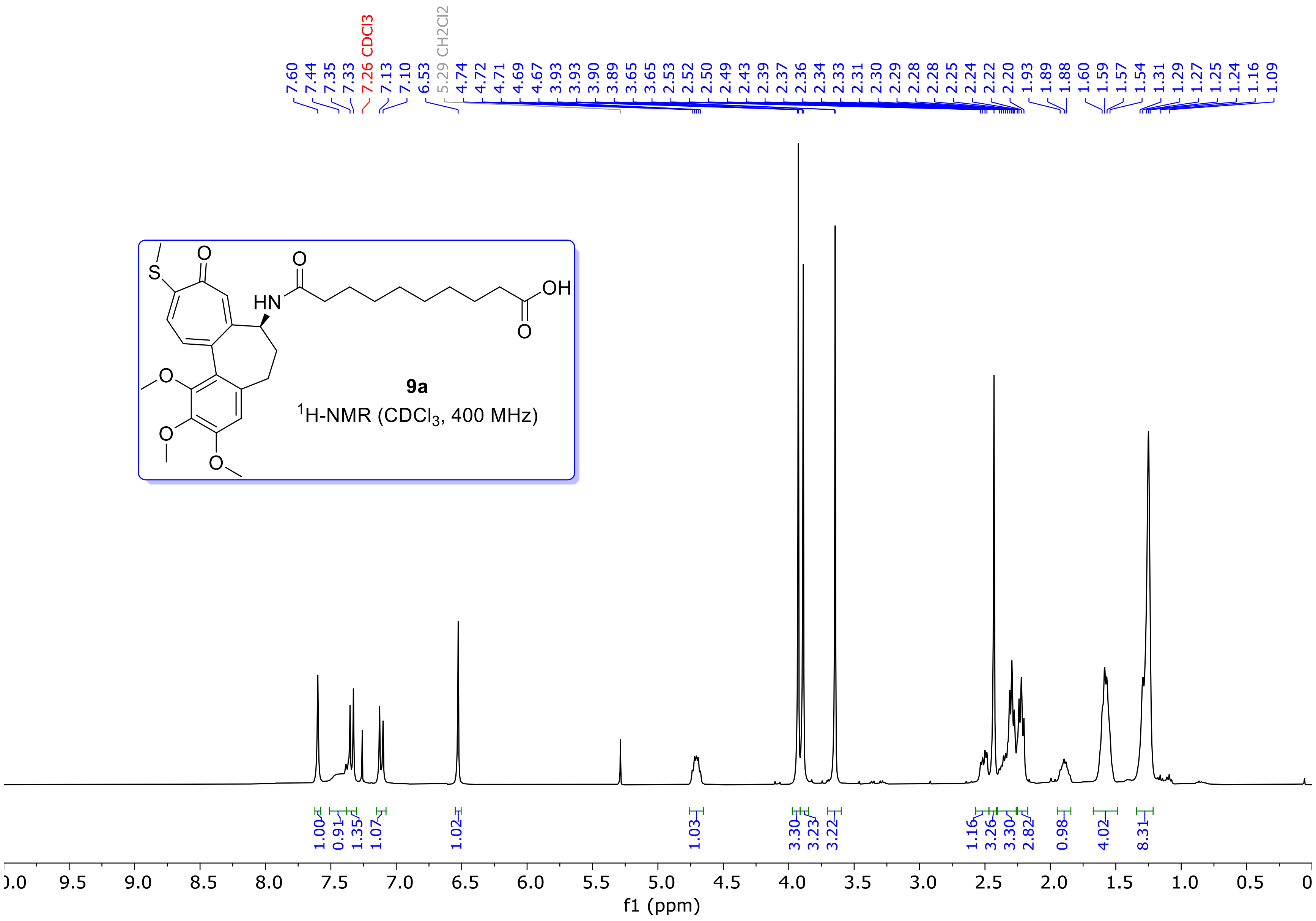
7.60
7.44
7.35
7.33
7.26 CDCl3
7.13
7.10
6.53
5.29 CH2Cl2
4.74
4.72
4.71
4.69
4.67
3.93
3.93
3.90
3.89
3.65
3.65
2.53
2.52
2.50
2.49
2.43
2.39
2.37
2.36
2.34
2.33
2.31
2.30
2.29
2.28
2.28
2.25
2.24
2.22
2.20
1.93
1.89
1.88
1.60
1.59
1.57
1.54
1.31
1.29
1.27
1.25
1.24
1.16
1.09
9a
1H-NMR (CDCl3, 400 MHz)
1.00
0.91
1.35
1.07
1.02
1.03
3.30
3.23
3.22
1.16
3.26
3.30
2.82
0.98
4.02
8.31
f1 (ppm)

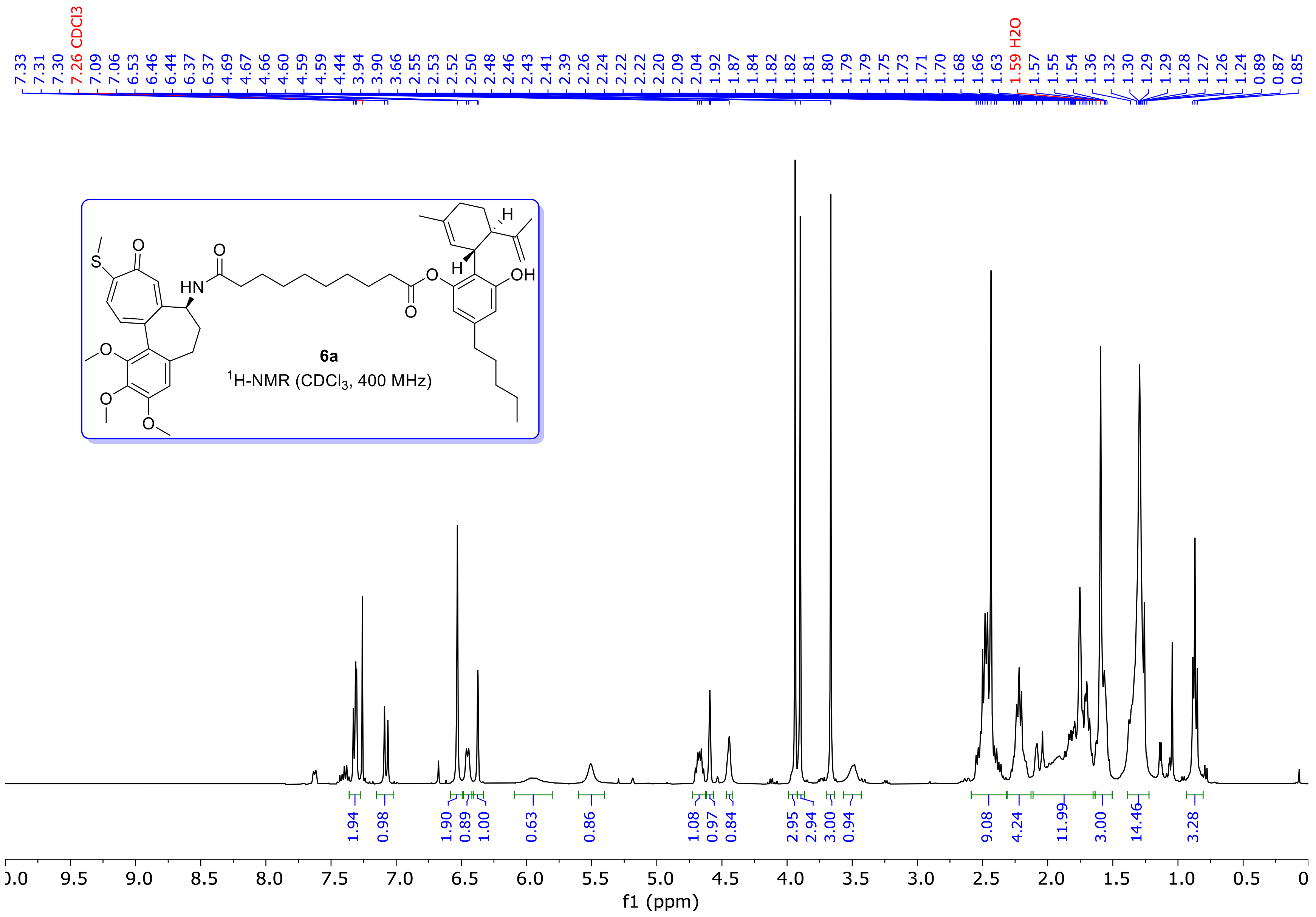
6a
¹H-NMR (CDCl₃, 400 MHz)
f1 (ppm)

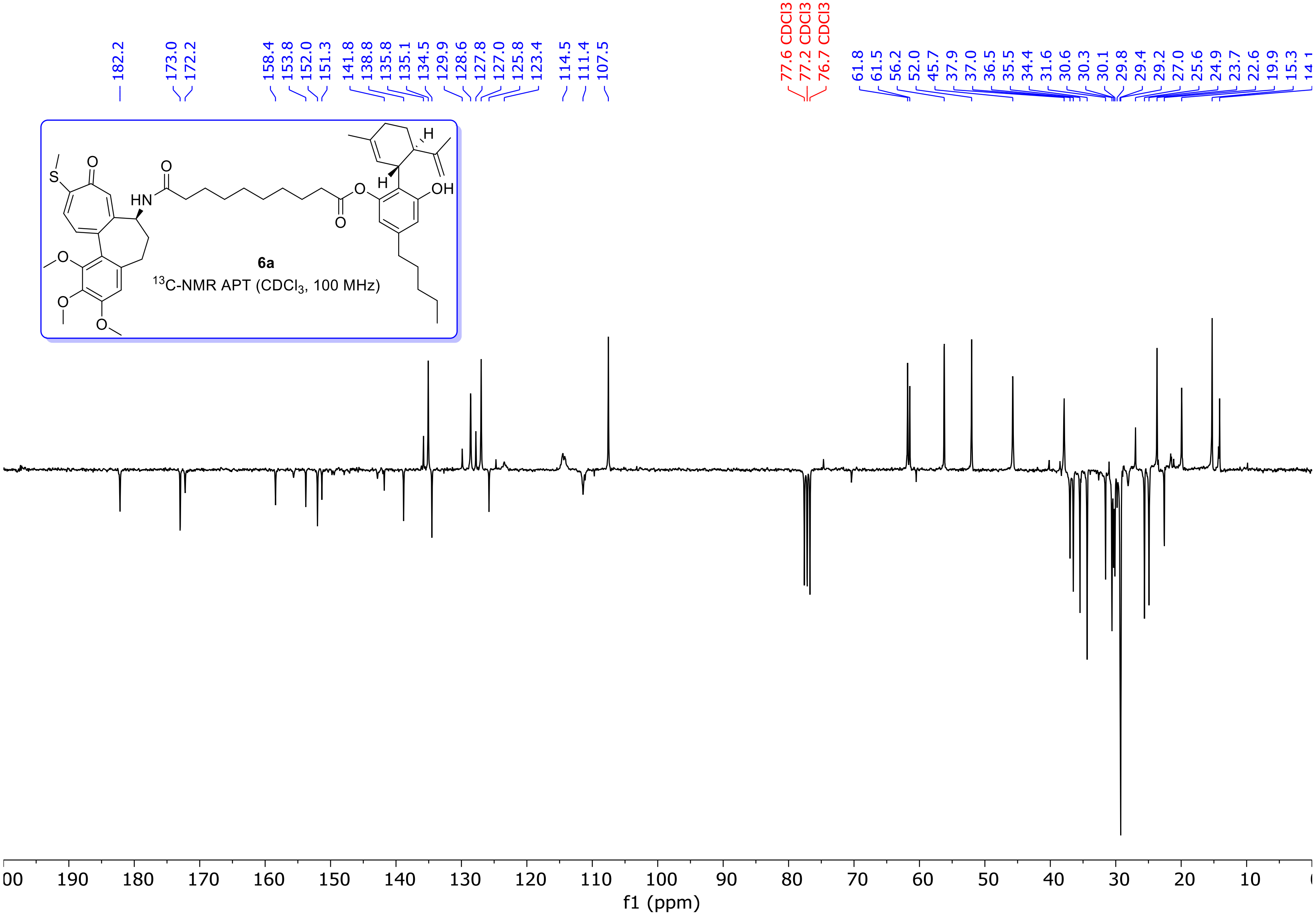
182.2
173.0
172.2
158.4
153.8
152.0
151.3
141.8
138.8
135.8
135.1
134.5
129.9
128.6
127.8
127.0
125.8
123.4
114.5
111.4
107.5
77.6 CDCl3
77.2 CDCl3
76.7 CDCl3
61.8
61.5
56.2
52.0
45.7
37.9
37.0
36.5
35.5
34.4
31.6
30.6
30.3
30.1
29.8
29.4
29.2
27.0
25.6
24.9
23.7
22.6
19.9
15.3
14.1
6a
^{13}C-NMR APT ($CDCl_3$, 100 MHz)
f1 (ppm)

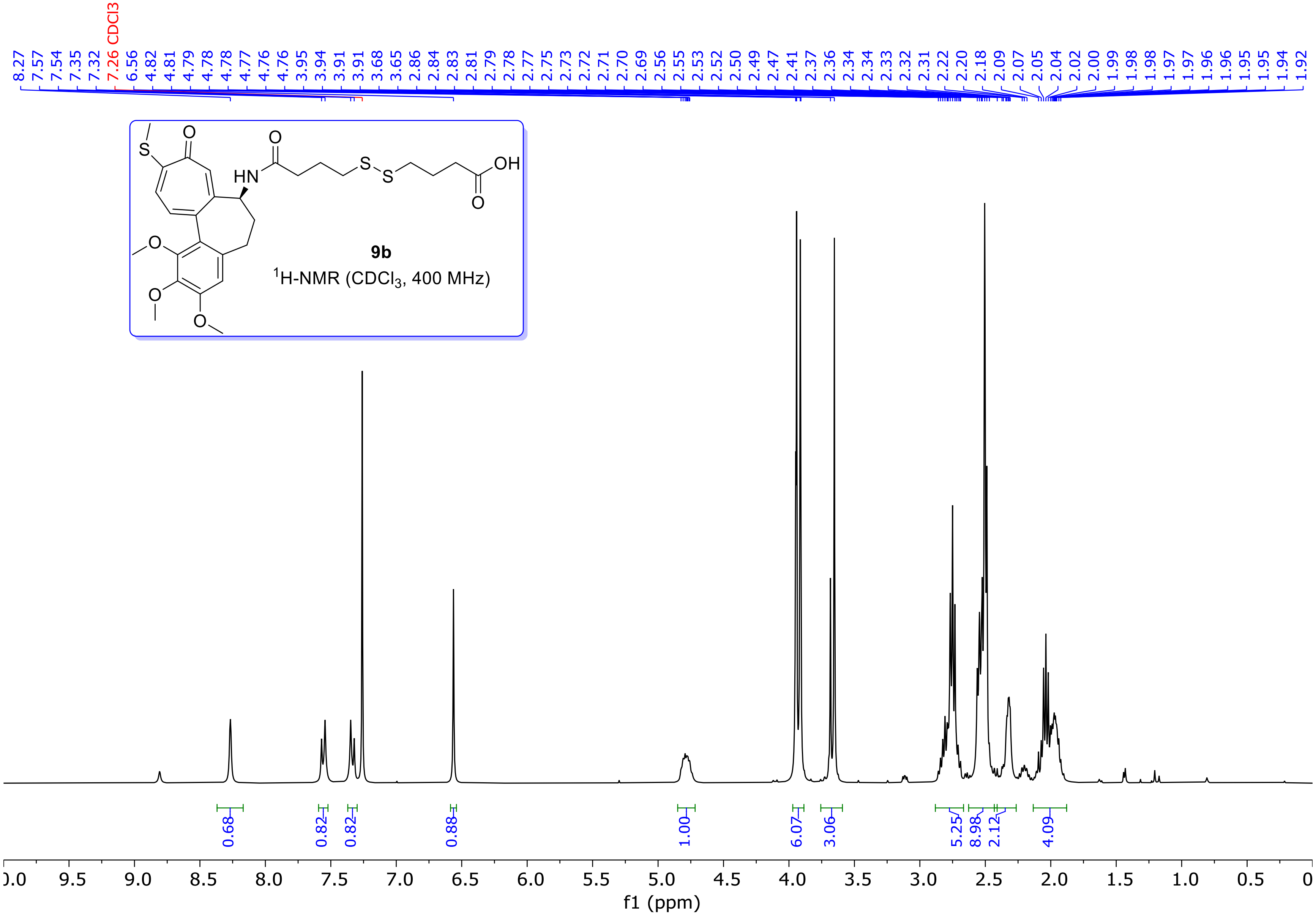
8.27 7.57 7.54 7.35 7.32 7.26 CDCl3 6.56 4.82 4.81 4.79 4.78 4.78 4.77 4.76 4.76 3.95 3.94 3.91 3.91 3.68 3.65 2.86 2.84 2.83 2.81 2.79 2.78 2.77 2.75 2.73 2.72 2.71 2.70 2.69 2.56 2.55 2.53 2.52 2.50 2.49 2.47 2.41 2.37 2.36 2.34 2.34 2.33 2.32 2.31 2.22 2.20 2.18 2.09 2.07 2.05 2.04 2.02 2.00 1.99 1.98 1.98 1.97 1.97 1.96 1.96 1.95 1.95 1.94 1.92
S
O
HN
O
S
S
OH
O
O
O
O
9b
1H-NMR (CDCl3, 400 MHz)
0.68
0.82
0.82
0.88
1.00
6.07
3.06
5.25
8.98
2.12
4.09
f1 (ppm)

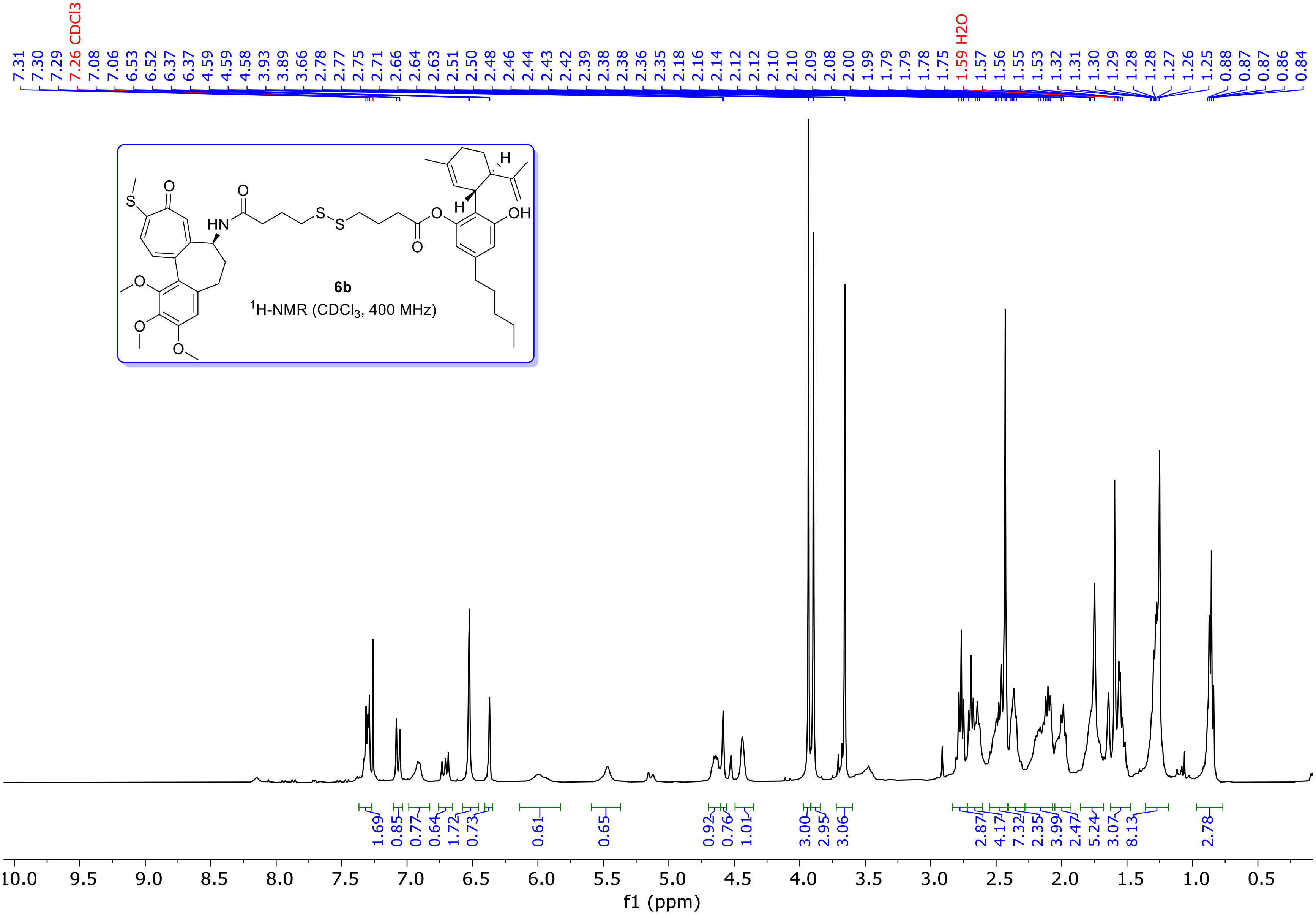
6b
^{1}H-NMR (CDCl3, 400 MHz)
7.26 CDCl3
1.59 H2O
f1 (ppm)

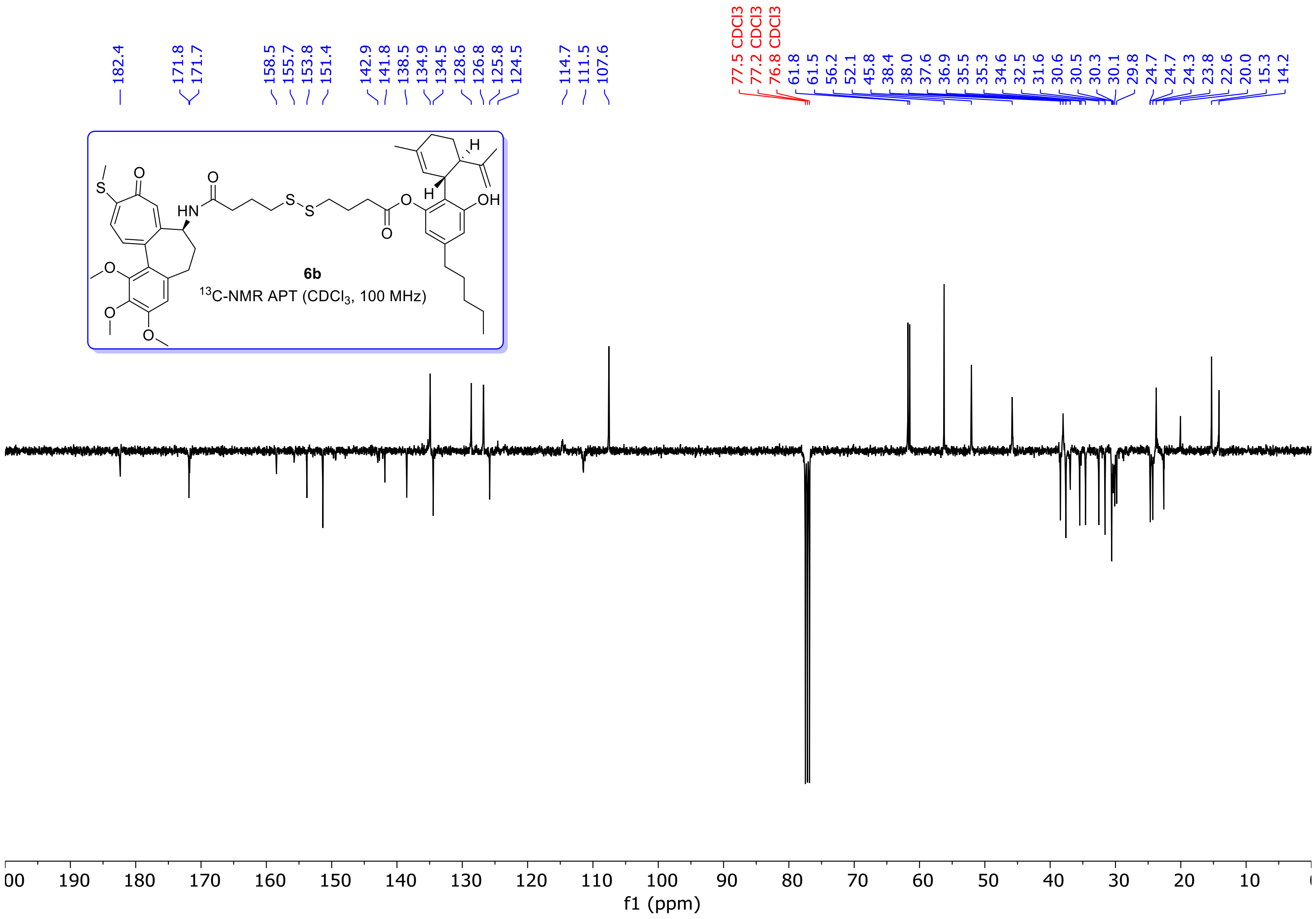
182.4
171.8
171.7
158.5
155.7
153.8
151.4
142.9
141.8
138.5
134.9
134.5
128.6
126.8
125.8
124.5
114.7
111.5
107.6
77.5 CDCl3
77.2 CDCl3
76.8 CDCl3
61.8
61.5
56.2
52.1
45.8
38.4
38.0
37.6
36.9
35.5
35.3
34.6
32.5
31.6
30.6
30.5
30.3
30.1
29.8
24.7
24.7
24.3
23.8
22.6
20.0
15.3
14.2
6b
13C-NMR APT (CDCl3, 100 MHz)
f1 (ppm)

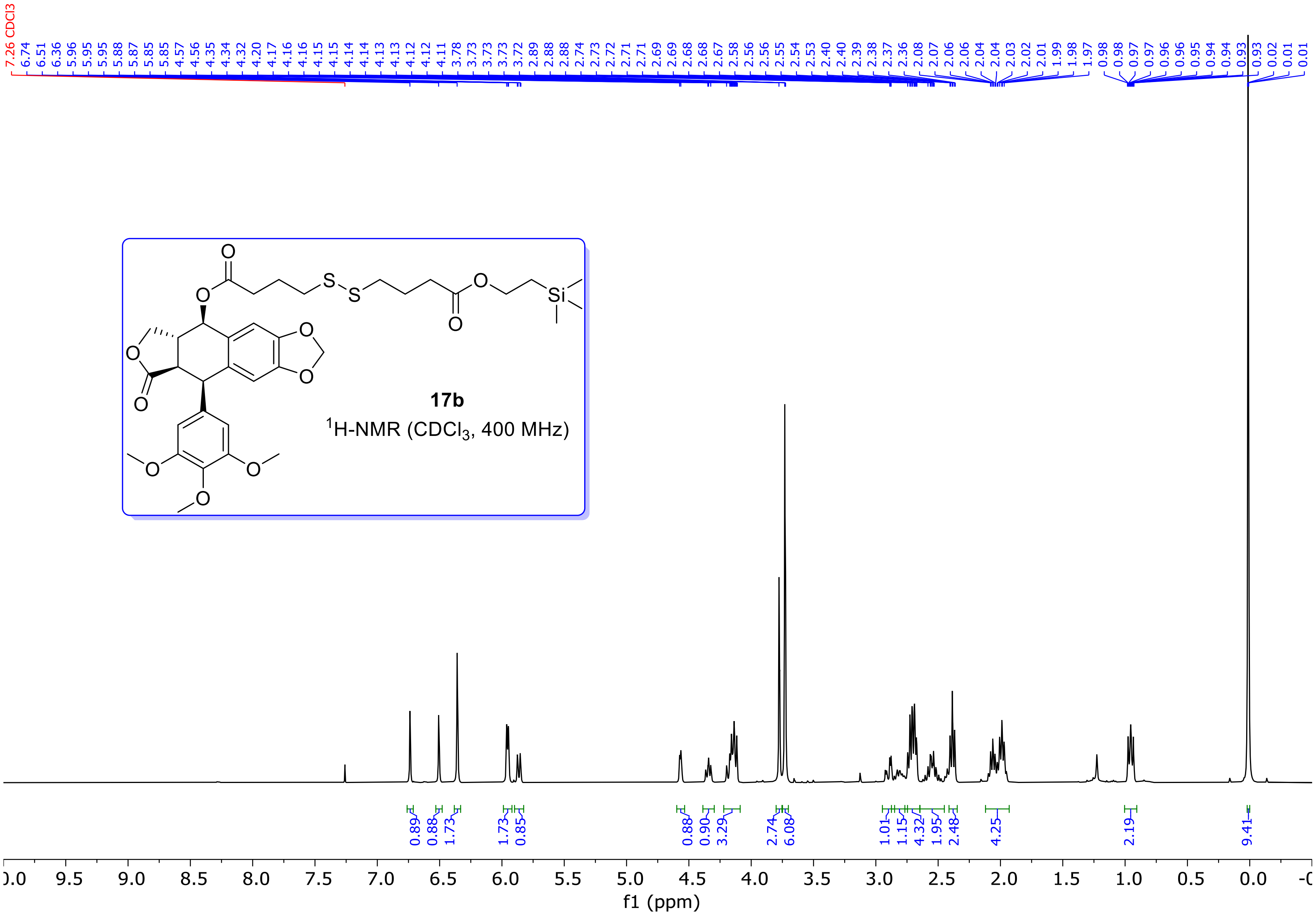
7.26 CDCl3
6.74 6.51 6.36 5.96 5.95 5.95 5.88 5.87 5.85 5.85 4.57 4.56 4.35 4.34 4.32 4.20 4.17 4.16 4.16 4.15 4.15 4.14 4.14 4.13 4.13 4.12 4.12 4.11 3.78 3.73 3.73 3.73 3.72 2.89 2.88 2.88 2.74 2.73 2.72 2.71 2.71 2.69 2.69 2.68 2.68 2.67 2.58 2.56 2.56 2.55 2.54 2.53 2.40 2.40 2.39 2.38 2.37 2.36 2.08 2.07 2.06 2.06 2.04 2.04 2.03 2.02 2.01 1.99 1.98 1.97 0.98 0.98 0.97 0.97 0.96 0.96 0.95 0.94 0.94 0.93 0.93 0.02 0.01 0.01
17b
1H-NMR (CDCl3, 400 MHz)
0.89 0.88 1.73 1.73 0.85 0.88 0.90 3.29 2.74 6.08 1.01 1.15 4.32 1.95 2.48 4.25 2.19 9.41
f1 (ppm)

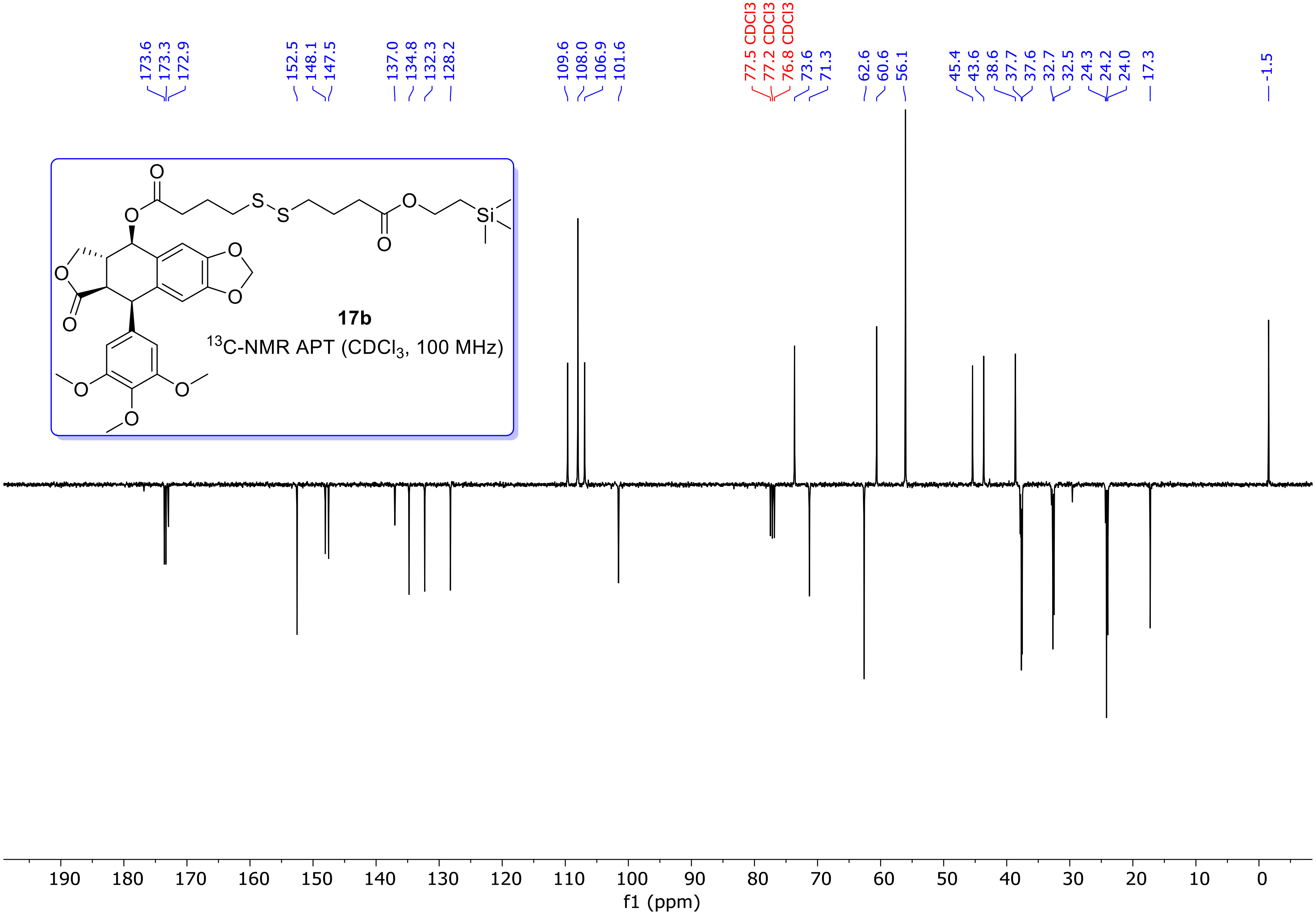
173.6
173.3
172.9
152.5
148.1
147.5
137.0
134.8
132.3
128.2
109.6
108.0
106.9
101.6
77.5 CDCl3
77.2 CDCl3
76.8 CDCl3
73.6
71.3
62.6
60.6
56.1
45.4
43.6
38.6
37.7
37.6
32.7
32.5
24.3
24.2
24.0
17.3
-1.5
17b
^{13}C-NMR APT ($CDCl_3$, 100 MHz)
190 180 170 160 150 140 130 120 110 100 90 80 70 60 50 40 30 20 10 0
f1 (ppm)

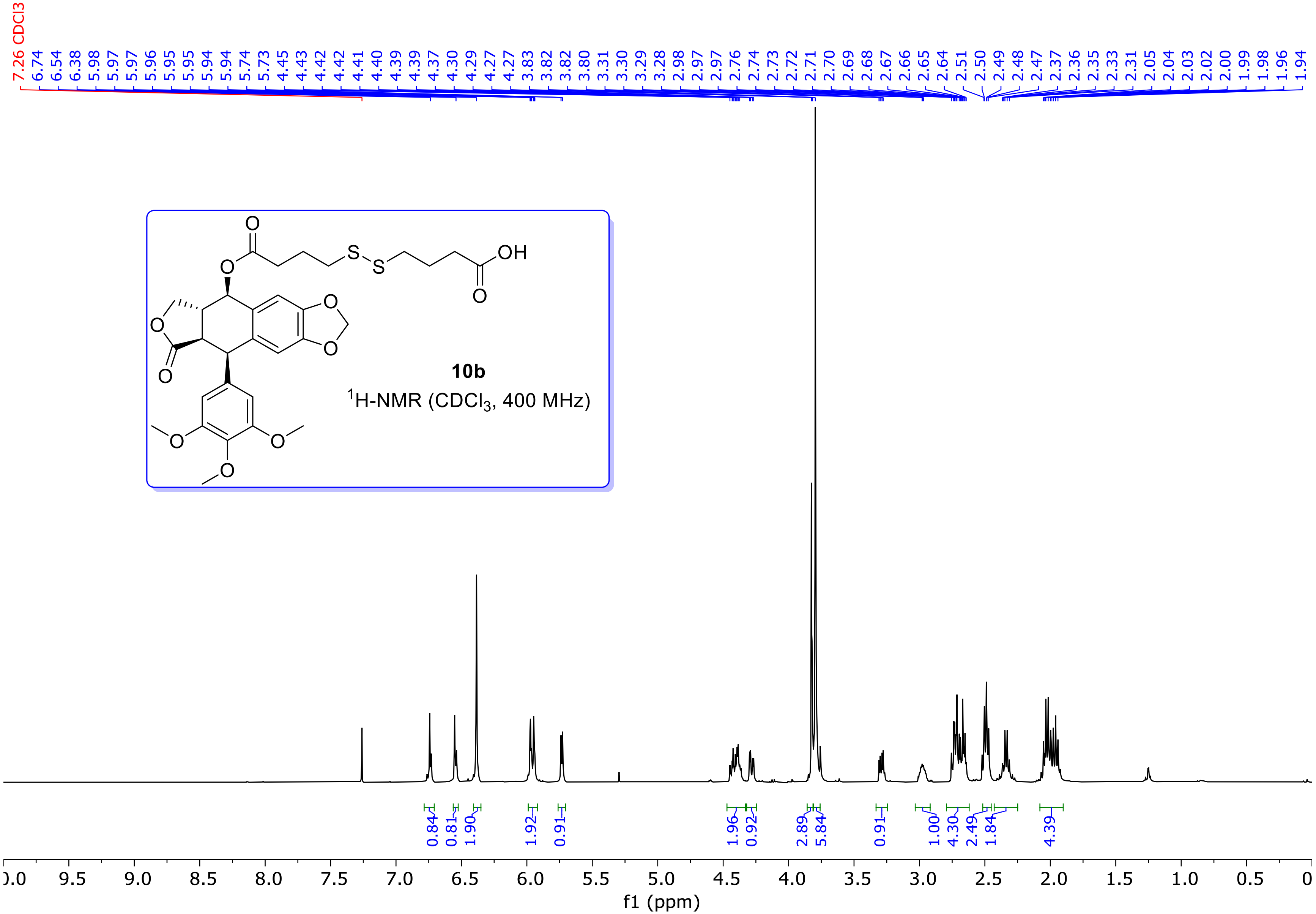
7.26 CDCl3
6.74 6.54 6.38 5.98 5.97 5.97 5.96 5.95 5.95 5.94 5.94 5.74 5.73 4.45 4.43 4.42 4.42 4.41 4.40 4.39 4.39 4.37 4.30 4.29 4.27 4.27 3.83 3.82 3.82 3.80 3.31 3.30 3.29 3.28 2.98 2.97 2.97 2.76 2.74 2.73 2.72 2.71 2.70 2.69 2.68 2.67 2.66 2.65 2.64 2.51 2.50 2.49 2.48 2.47 2.37 2.36 2.35 2.33 2.31 2.05 2.04 2.03 2.02 2.00 1.99 1.98 1.96 1.94
O
O
S
S
OH
O
O
O
O
O
O
O
O
O
10b
^{1}H-NMR (CDCl$_3$, 400 MHz)
0.84 0.81 1.90 1.92 0.91 1.96 0.92 2.89 5.84 0.91 1.00 4.30 2.49 1.84 4.39
9.5 9.0 8.5 8.0 7.5 7.0 6.5 6.0 5.5 5.0 4.5 4.0 3.5 3.0 2.5 2.0 1.5 1.0 0.5 0
f1 (ppm)

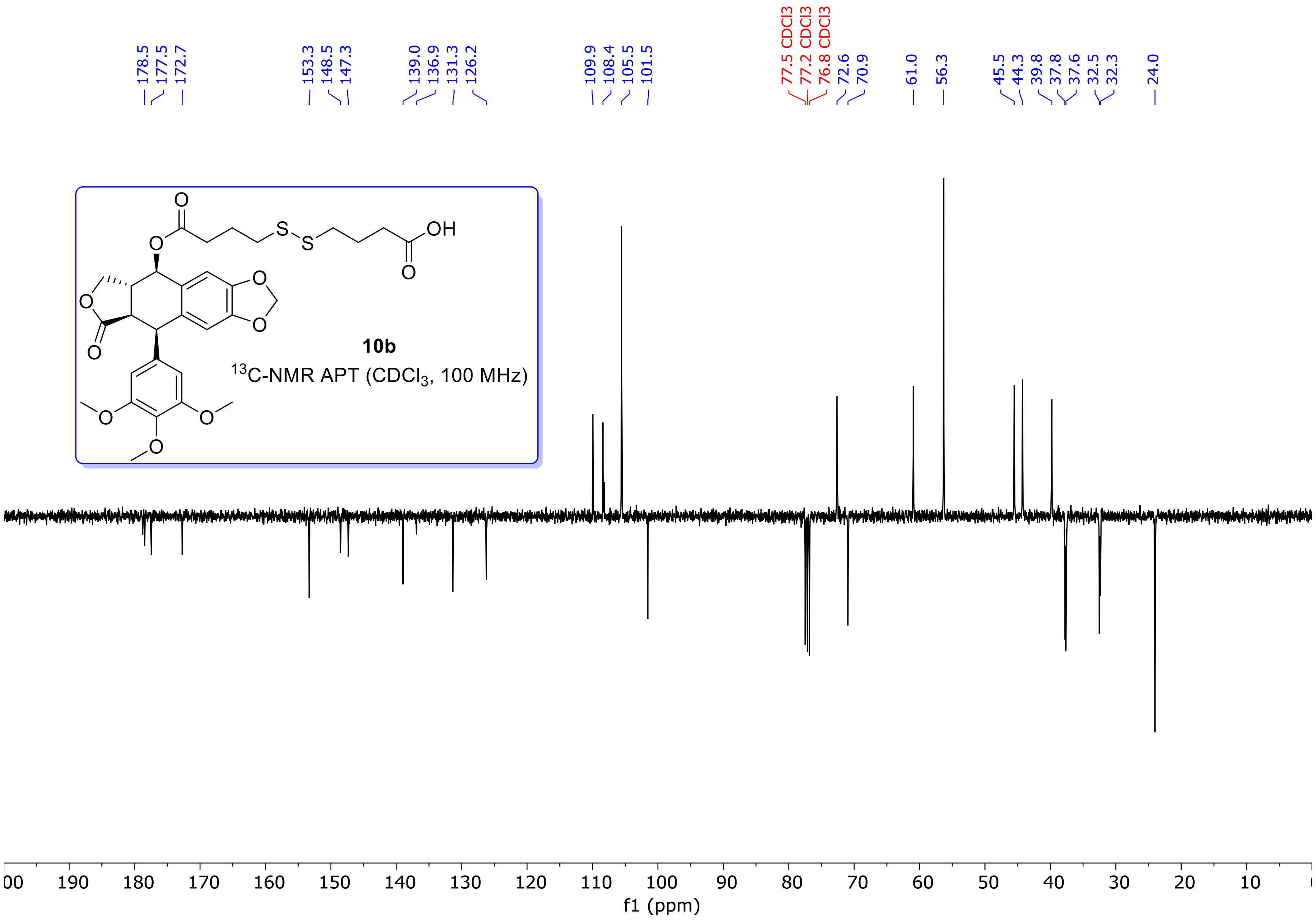

178.5
177.5
172.7
153.3
148.5
147.3
139.0
136.9
131.3
126.2
109.9
108.4
105.5
101.5
77.5 CDCl3
77.2 CDCl3
76.8 CDCl3
72.6
70.9
61.0
56.3
45.5
44.3
39.8
37.8
37.6
32.5
32.3
24.0
10b
13C-NMR APT (CDCl3, 100 MHz)
f1 (ppm)

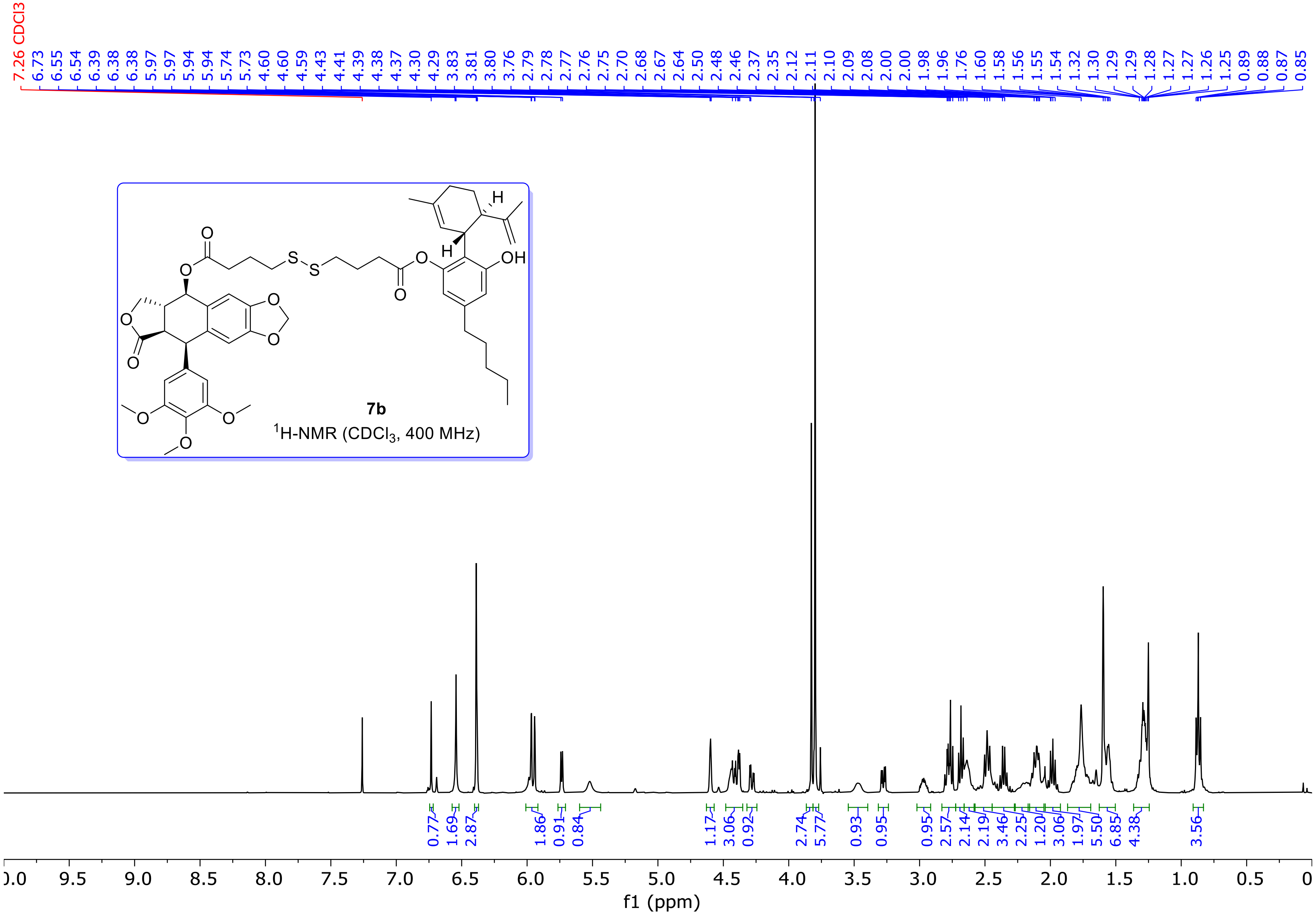
7.26 CDCl3
6.73
6.55
6.54
6.39
6.38
6.38
5.97
5.97
5.94
5.94
5.74
5.73
4.60
4.60
4.59
4.43
4.41
4.39
4.38
4.37
4.30
4.29
3.83
3.81
3.80
3.76
2.79
2.78
2.77
2.76
2.75
2.70
2.68
2.67
2.64
2.50
2.48
2.46
2.37
2.35
2.12
2.11
2.10
2.09
2.08
2.00
2.00
1.98
1.96
1.76
1.60
1.58
1.56
1.55
1.54
1.32
1.30
1.29
1.29
1.28
1.27
1.27
1.26
1.25
0.89
0.88
0.87
0.85
O
O
S
S
O
O
OH
H
H
O
O
O
O
O
O
O
O
7b
1H-NMR (CDCl3, 400 MHz)
0.77
1.69
2.87
1.86
0.91
0.84
1.17
3.06
0.92
2.74
5.77
0.93
0.95
0.95
2.57
2.14
2.19
3.46
2.25
1.20
3.06
1.97
5.50
6.85
4.38
3.56
.0
9.5
9.0
8.5
8.0
7.5
7.0
6.5
6.0
5.5
5.0
4.5
4.0
3.5
3.0
2.5
2.0
1.5
1.0
0.5
0
f1 (ppm)

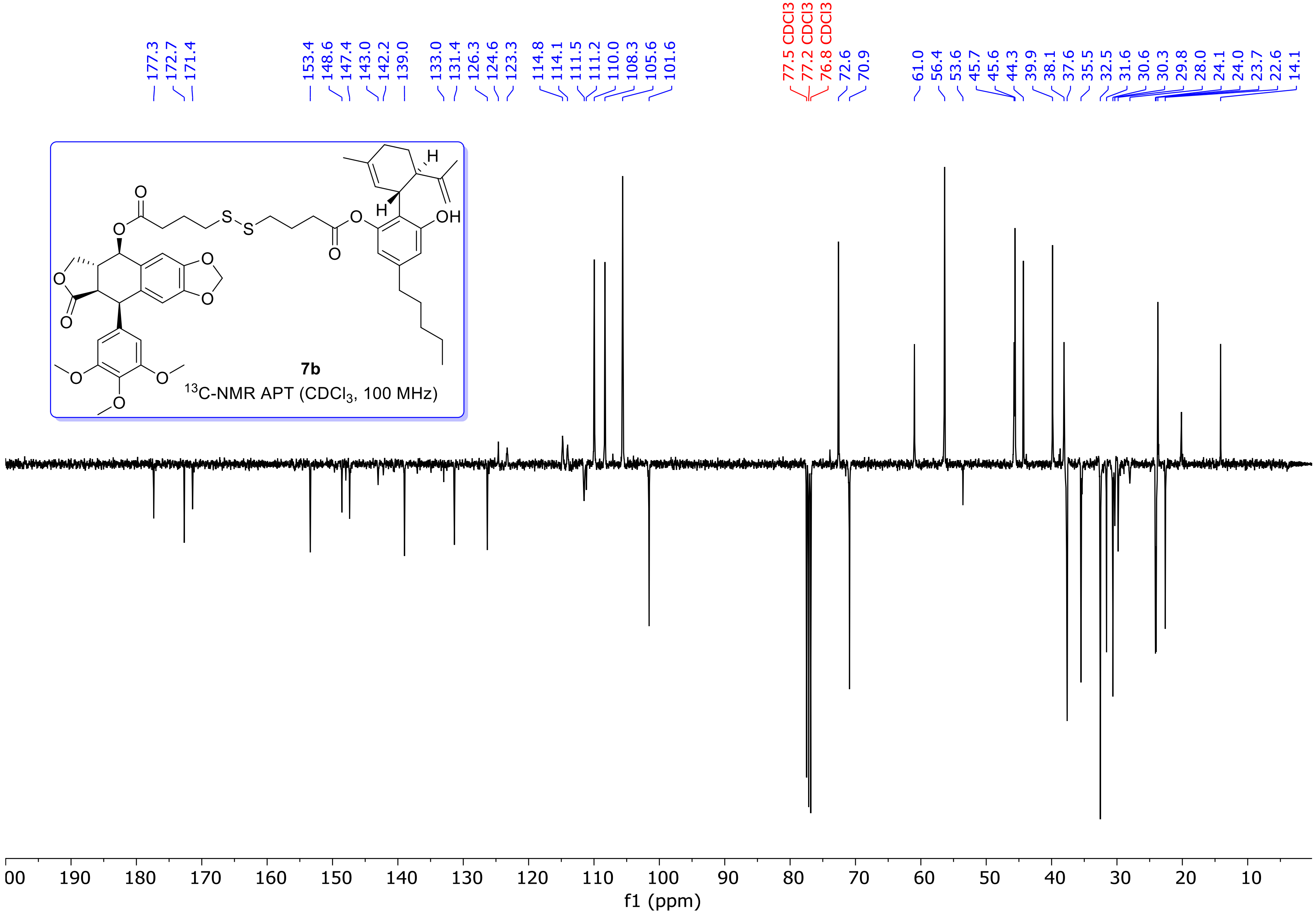
177.3
172.7
171.4
153.4
148.6
147.4
143.0
142.2
139.0
133.0
131.4
126.3
124.6
123.3
114.8
114.1
111.5
111.2
110.0
108.3
105.6
101.6
77.5 CDCl3
77.2 CDCl3
76.8 CDCl3
72.6
70.9
61.0
56.4
53.6
45.7
45.6
44.3
39.9
38.1
37.6
35.5
32.5
31.6
30.6
30.3
29.8
28.0
24.1
24.0
23.7
22.6
14.1
7b
13C-NMR APT (CDCl3, 100 MHz)
f1 (ppm)

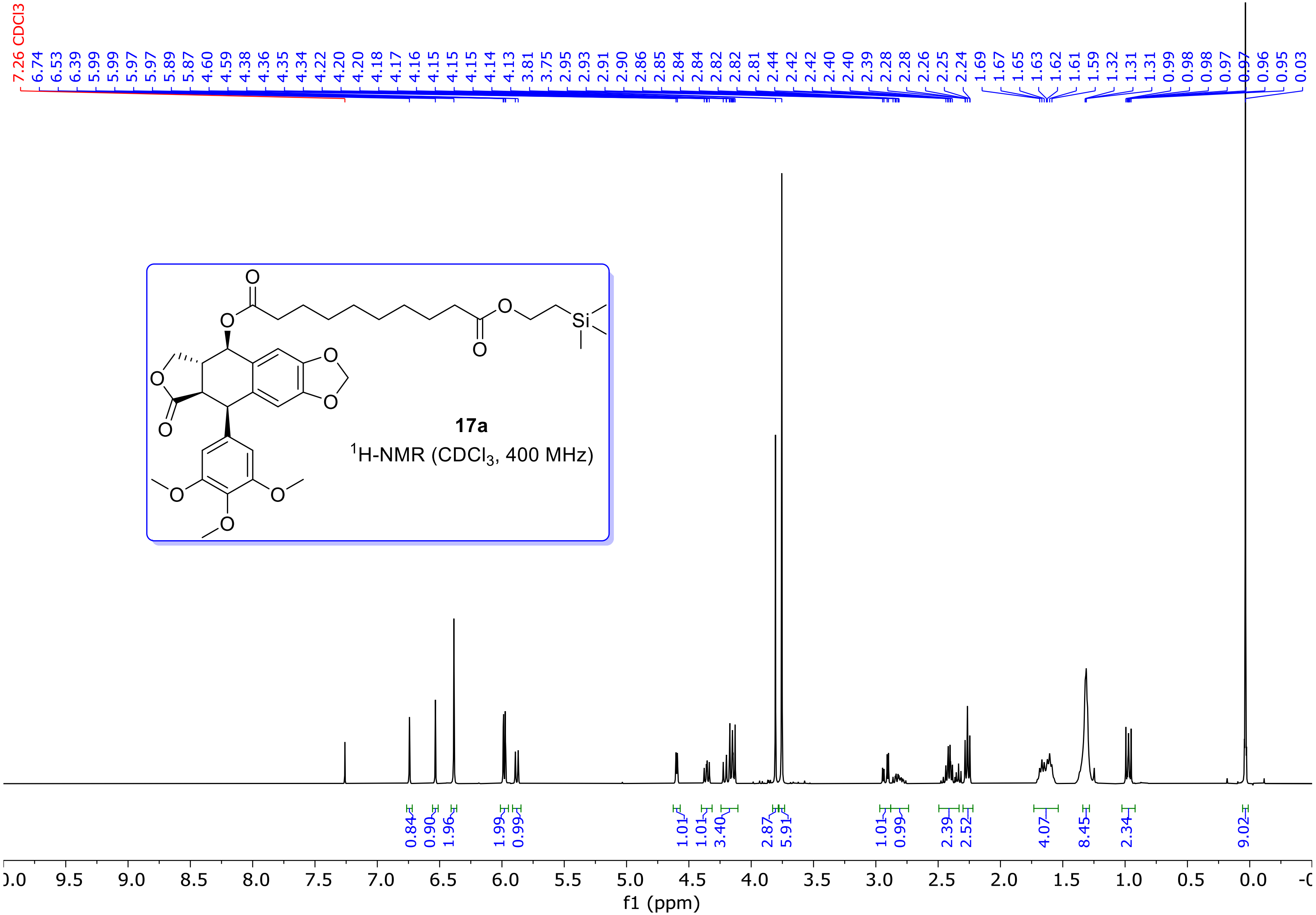
7.26 CDCl3
17a
^{1}H-NMR (CDCl$_3$, 400 MHz)
f1 (ppm)

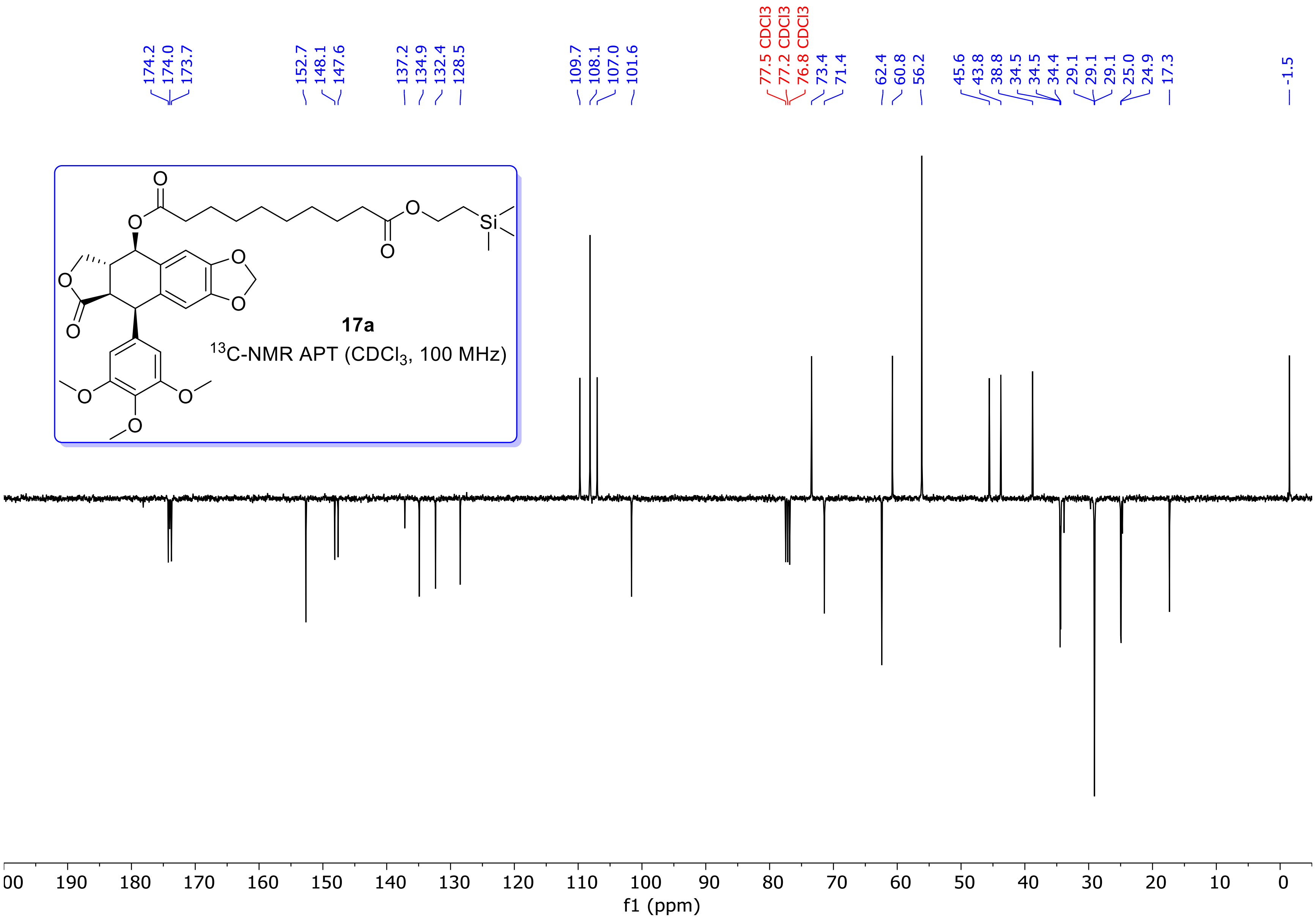

174.2
174.0
173.7
152.7
148.1
147.6
137.2
134.9
132.4
128.5
109.7
108.1
107.0
101.6
77.5 CDCl3
77.2 CDCl3
76.8 CDCl3
73.4
71.4
62.4
60.8
56.2
45.6
43.8
38.8
34.5
34.5
34.4
29.1
29.1
29.1
25.0
24.9
17.3
-1.5
17a
13C-NMR APT (CDCl3, 100 MHz)
00 190 180 170 160 150 140 130 120 110 100 90 80 70 60 50 40 30 20 10 0
f1 (ppm)

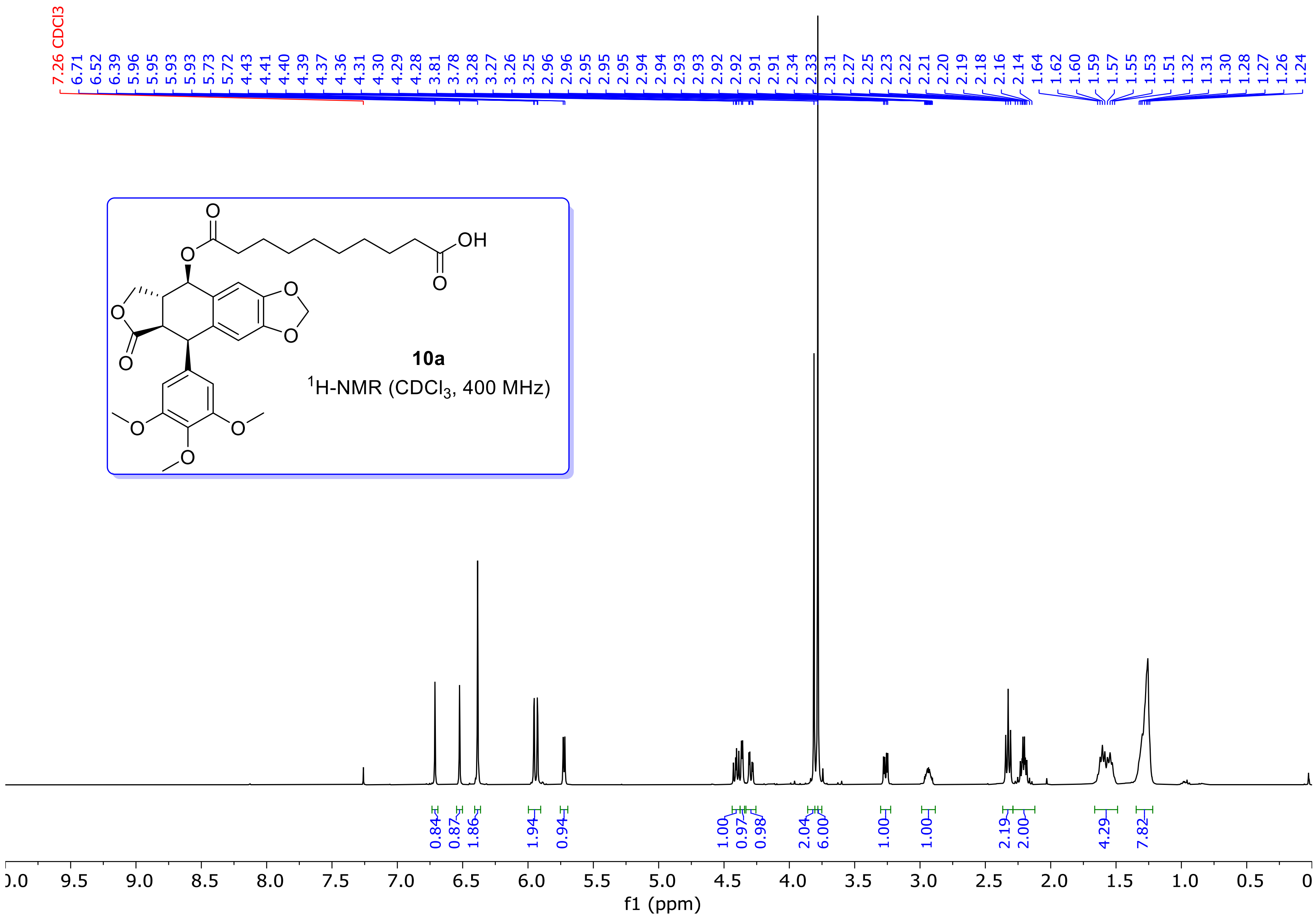

7.26 CDCl3
6.71 6.52 6.39 5.96 5.95 5.93 5.93 5.73 5.72 4.43 4.41 4.40 4.39 4.37 4.36 4.31 4.30 4.29 4.28 3.81 3.78 3.28 3.27 3.26 3.25 2.96 2.96 2.95 2.95 2.95 2.94 2.94 2.93 2.93 2.92 2.92 2.91 2.91 2.34 2.33 2.31 2.27 2.25 2.23 2.22 2.21 2.20 2.19 2.18 2.16 2.14 1.64 1.62 1.60 1.59 1.57 1.55 1.53 1.51 1.32 1.31 1.30 1.28 1.27 1.26 1.24
OH
10a
1H-NMR (CDCl3, 400 MHz)
0.84 0.87 1.86 1.94 0.94 1.00 0.97 0.98 2.04 6.00 1.00 1.00 2.19 2.00 4.29 7.82
10.0 9.5 9.0 8.5 8.0 7.5 7.0 6.5 6.0 5.5 5.0 4.5 4.0 3.5 3.0 2.5 2.0 1.5 1.0 0.5 0
f1 (ppm)

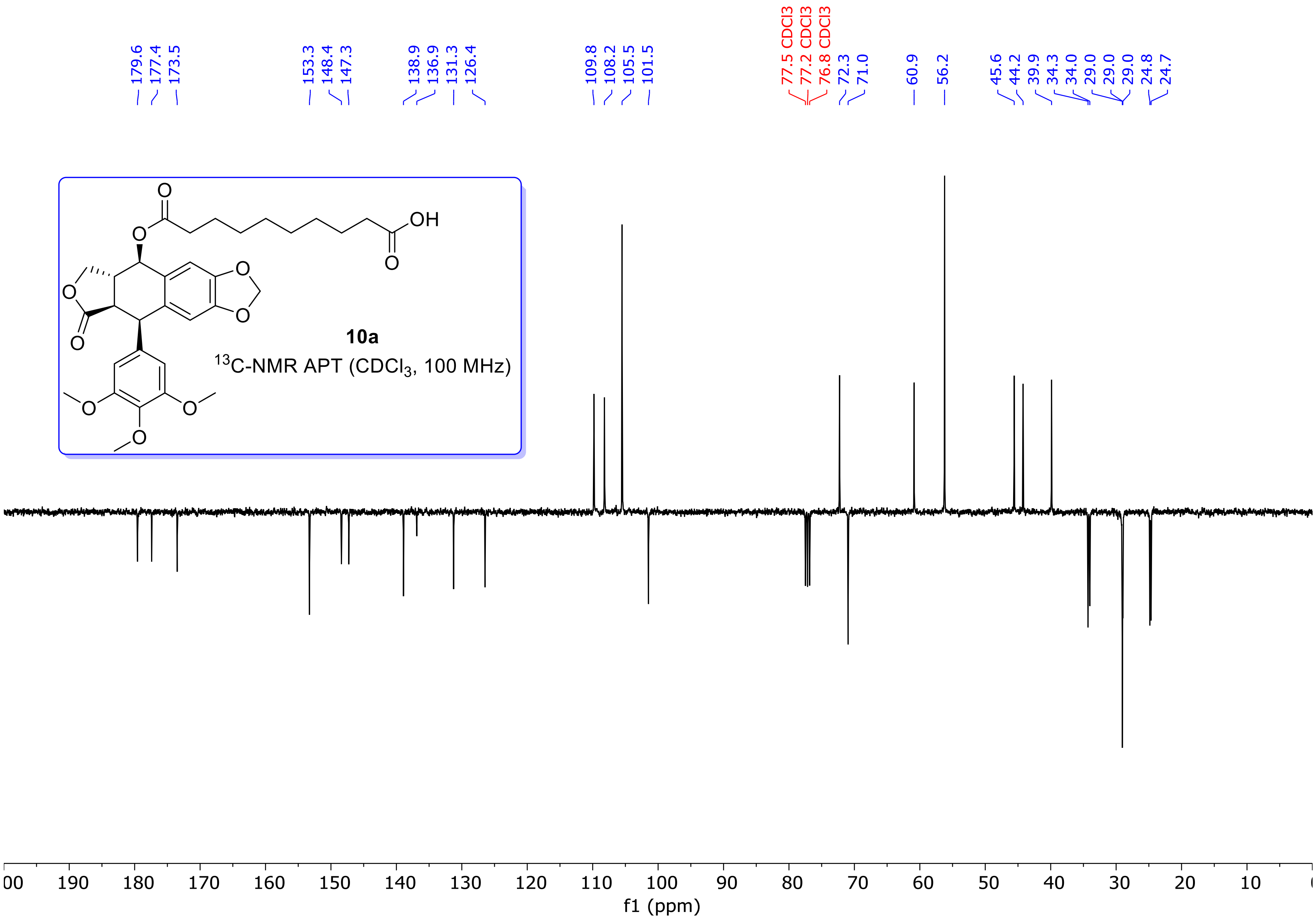
179.6
177.4
173.5
153.3
148.4
147.3
138.9
136.9
131.3
126.4
109.8
108.2
105.5
101.5
77.5 CDCl3
77.2 CDCl3
76.8 CDCl3
72.3
71.0
60.9
56.2
45.6
44.2
39.9
34.3
34.0
29.0
29.0
29.0
24.8
24.7
O
OH
O
O
O
O
O
O
O
O
O
O
10a
13C-NMR APT (CDCl3, 100 MHz)
190
180
170
160
150
140
130
120
110
100
90
80
70
60
50
40
30
20
10
f1 (ppm)

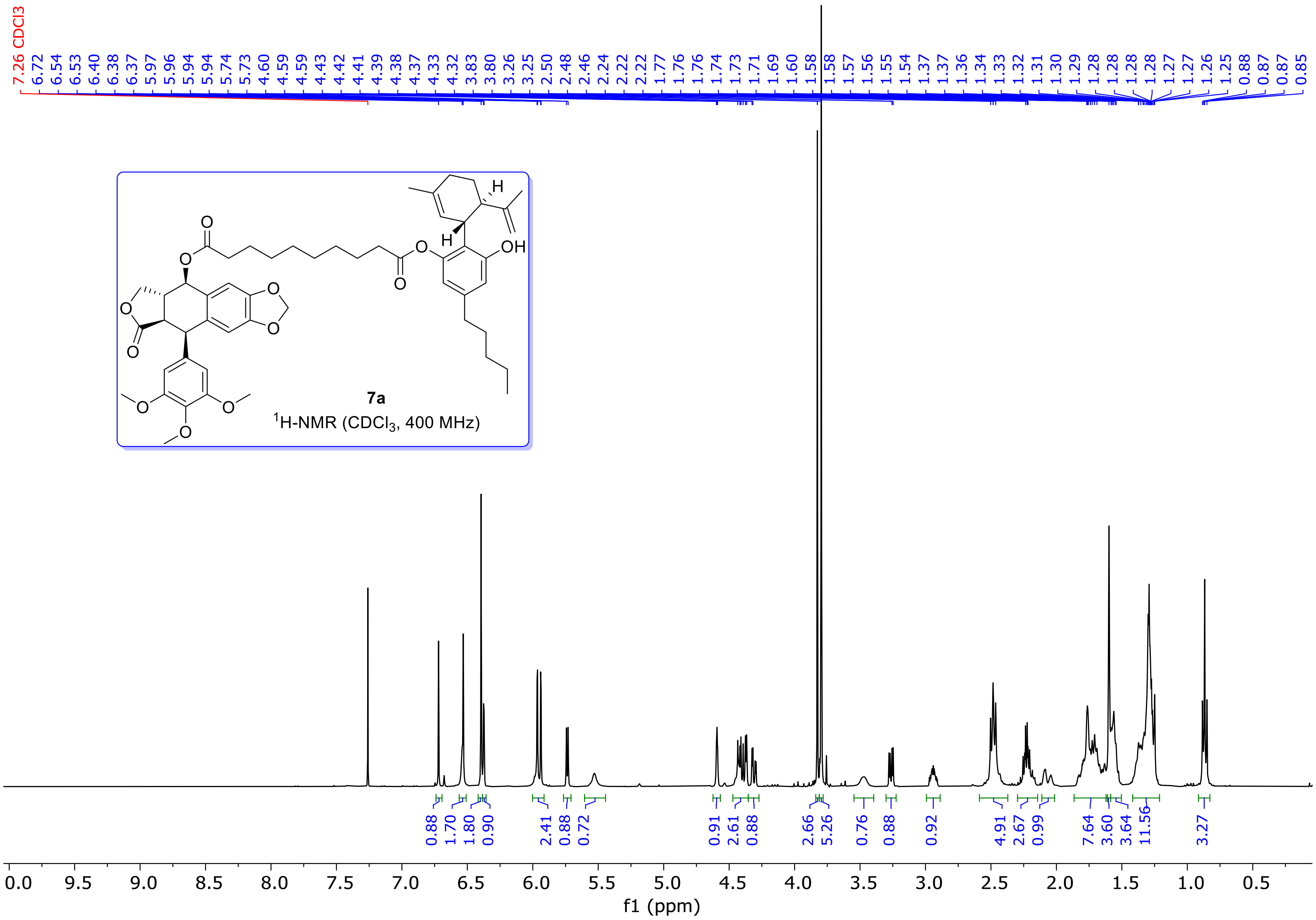
7.26 CDCl3
6.72 6.54 6.53 6.40 6.38 6.37 5.97 5.96 5.94 5.94 5.74 5.73 4.60 4.59 4.59 4.43 4.42 4.41 4.39 4.38 4.37 4.33 4.32 3.83 3.80 3.26 3.25 2.50 2.48 2.46 2.24 2.22 2.22 1.77 1.76 1.76 1.74 1.73 1.71 1.69 1.60 1.58 1.58 1.57 1.56 1.55 1.54 1.37 1.37 1.36 1.34 1.33 1.32 1.31 1.30 1.29 1.28 1.28 1.28 1.28 1.27 1.27 1.26 1.25 0.88 0.87 0.87 0.85
7a
1H-NMR (CDCl3, 400 MHz)
0.88 1.70 1.80 0.90 2.41 0.88 0.72 0.91 2.61 0.88 2.66 5.26 0.76 0.88 0.92 4.91 2.67 0.99 7.64 3.60 3.64 11.56 3.27
0.0 9.5 9.0 8.5 8.0 7.5 7.0 6.5 6.0 5.5 5.0 4.5 4.0 3.5 3.0 2.5 2.0 1.5 1.0 0.5
f1 (ppm)

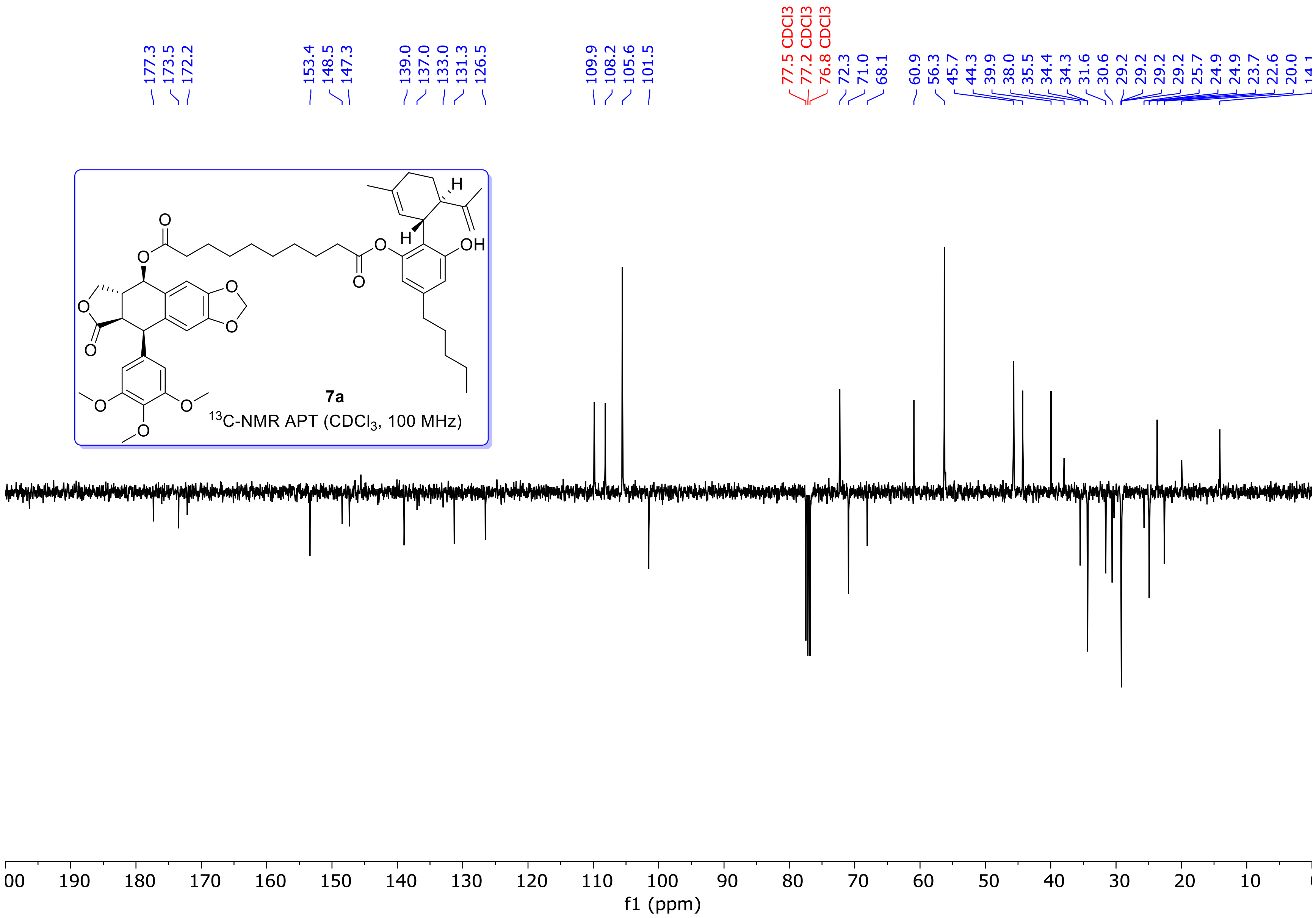

177.3
173.5
172.2
153.4
148.5
147.3
139.0
137.0
133.0
131.3
126.5
109.9
108.2
105.6
101.5
77.5 CDCl3
77.2 CDCl3
76.8 CDCl3
72.3
71.0
68.1
60.9
56.3
45.7
44.3
39.9
38.0
35.5
34.4
34.3
31.6
30.6
29.2
29.2
29.2
29.2
25.7
24.9
24.9
23.7
22.6
20.0
O
O
O
O
O
O
O
O
OH
H
H
7a
13C-NMR APT (CDCl3, 100 MHz)
190
180
170
160
150
140
130
120
110
100
90
80
70
60
50
40
30
20
10
f1 (ppm)

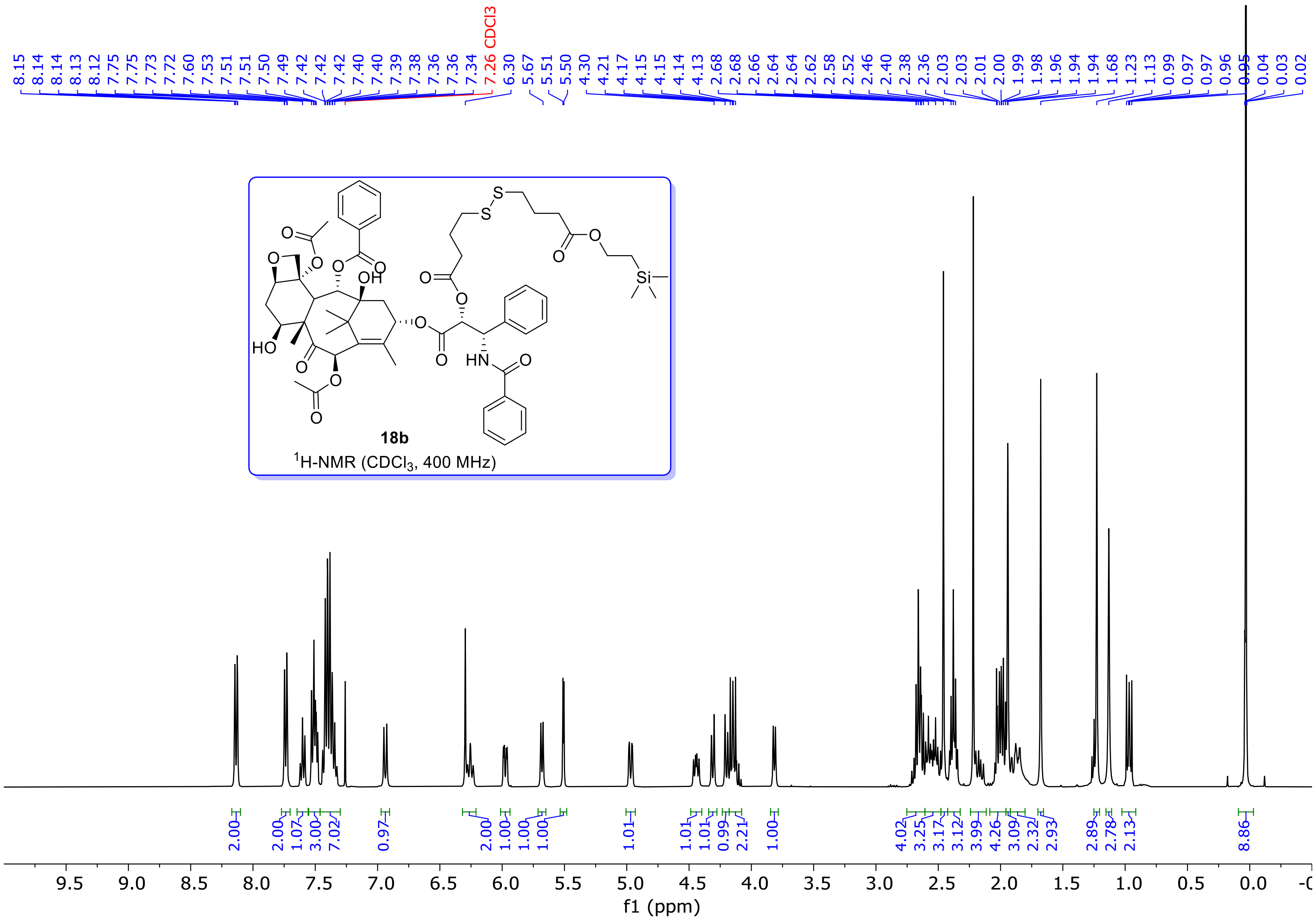

18b
^{1}H-NMR (CDCl$_3$, 400 MHz)
f1 (ppm)

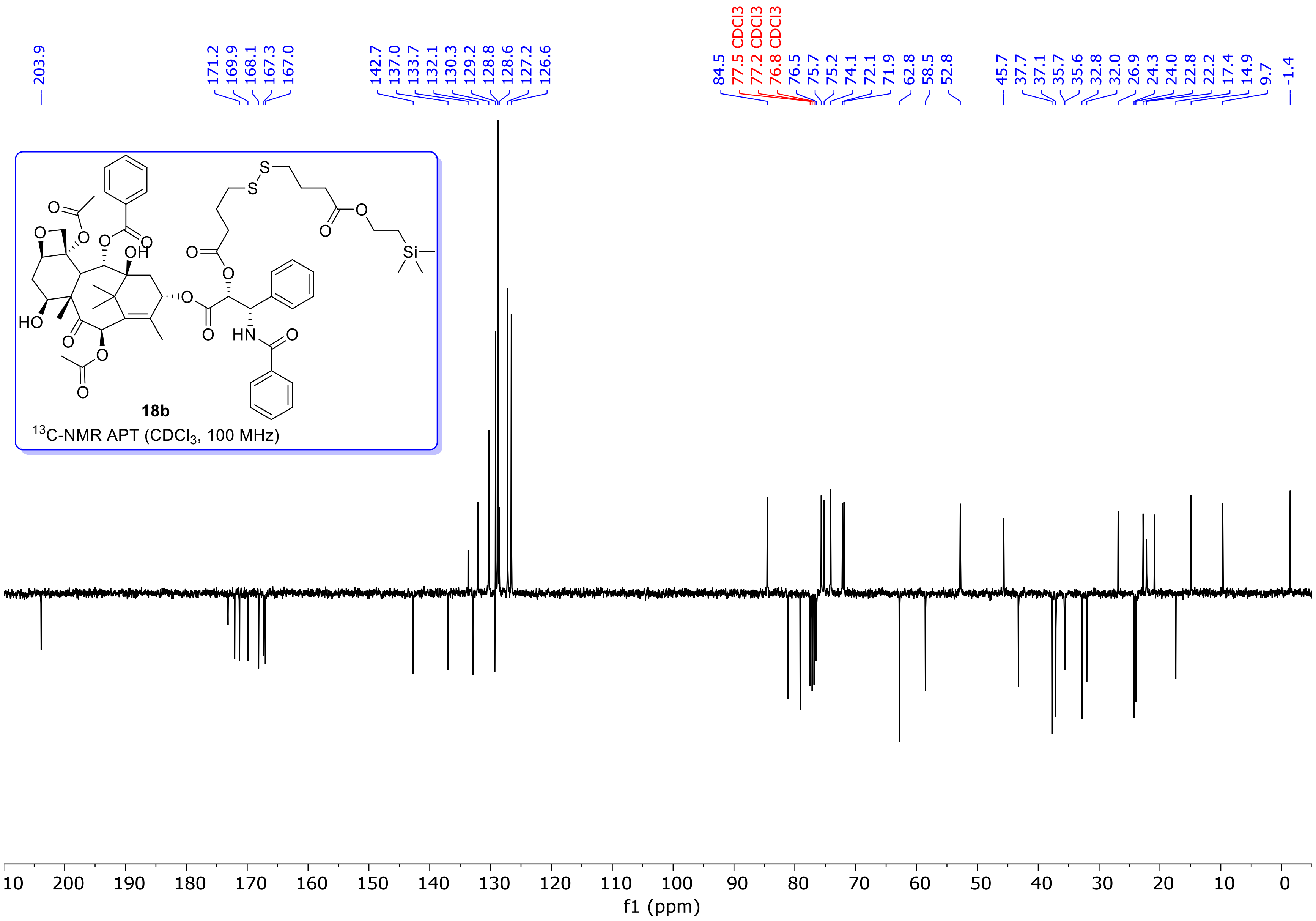
203.9
171.2
169.9
168.1
167.3
167.0
142.7
137.0
133.7
132.1
130.3
129.2
128.8
128.6
127.2
126.6
84.5
77.5 CDCl3
77.2 CDCl3
76.8 CDCl3
76.5
75.7
75.2
74.1
72.1
71.9
62.8
58.5
52.8
45.7
37.7
37.1
35.7
35.6
32.8
32.0
26.9
24.3
24.0
22.8
22.2
17.4
14.9
9.7
-1.4
18b
^{13}C-NMR APT ($CDCl_3$, 100 MHz)
210 200 190 180 170 160 150 140 130 120 110 100 90 80 70 60 50 40 30 20 10 0
f1 (ppm)

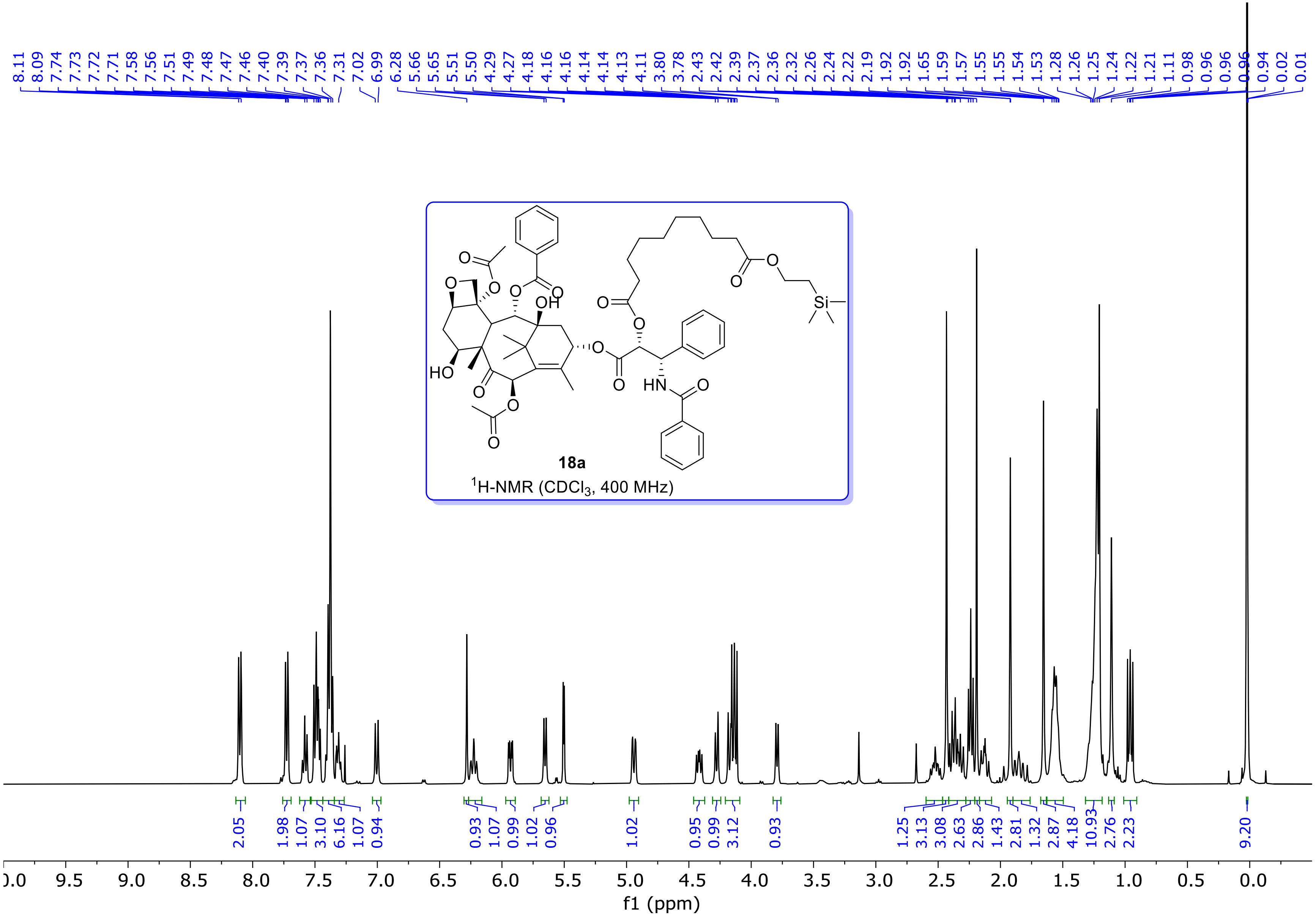
18a
1H-NMR (CDCl3, 400 MHz)
f1 (ppm)

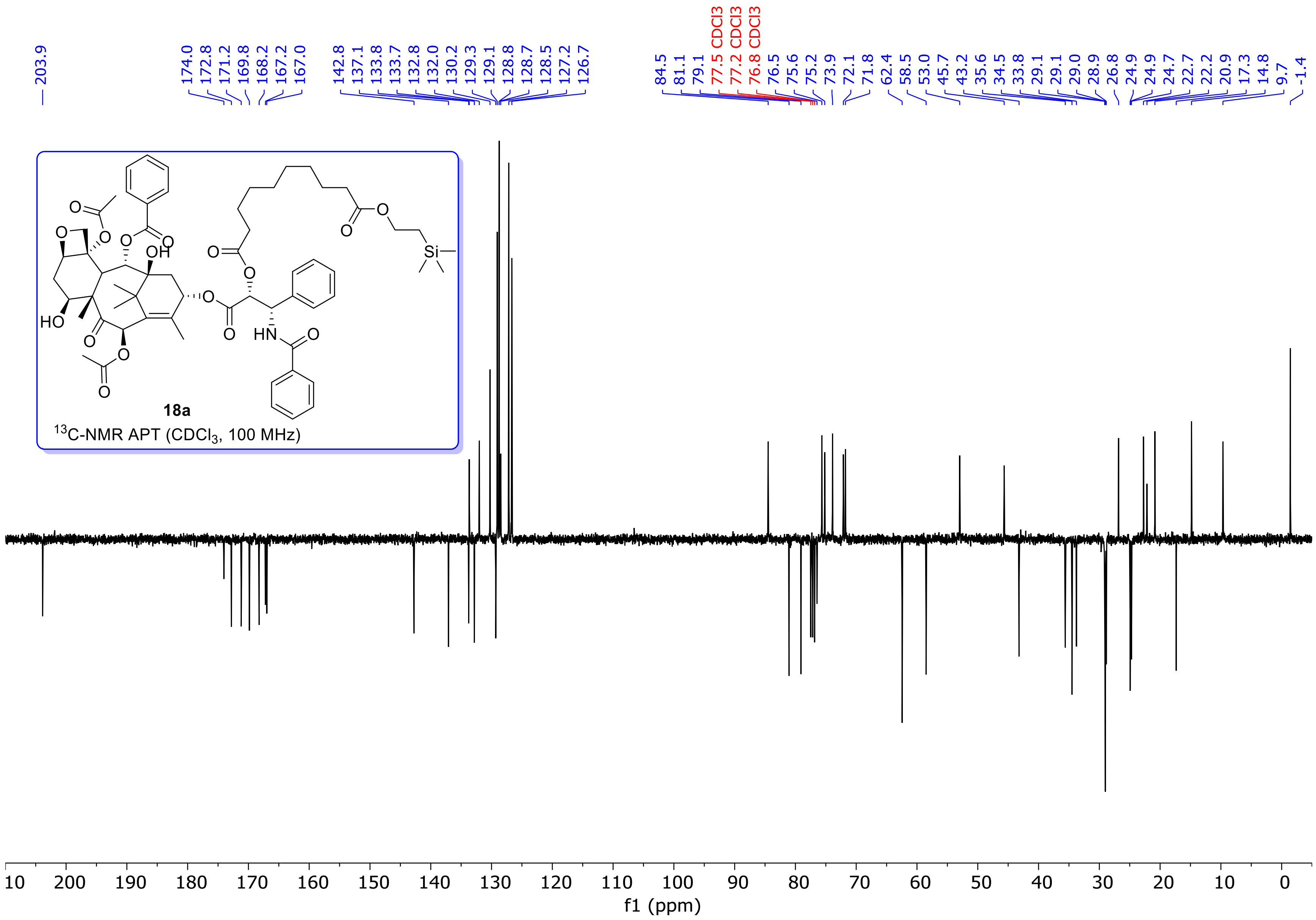
203.9
174.0
172.8
171.2
169.8
168.2
167.2
167.0
142.8
137.1
133.8
133.7
132.8
132.0
130.2
129.3
129.1
128.8
128.7
128.5
127.2
126.7
84.5
81.1
79.1
77.5 CDCl3
77.2 CDCl3
76.8 CDCl3
76.5
75.6
75.2
73.9
72.1
71.8
62.4
58.5
53.0
45.7
43.2
35.6
34.5
33.8
29.1
29.1
29.0
28.9
26.8
24.9
24.9
24.7
22.7
22.2
20.9
17.3
14.8
9.7
-1.4
18a
^{13}C-NMR APT ($CDCl_3$, 100 MHz)
f1 (ppm)

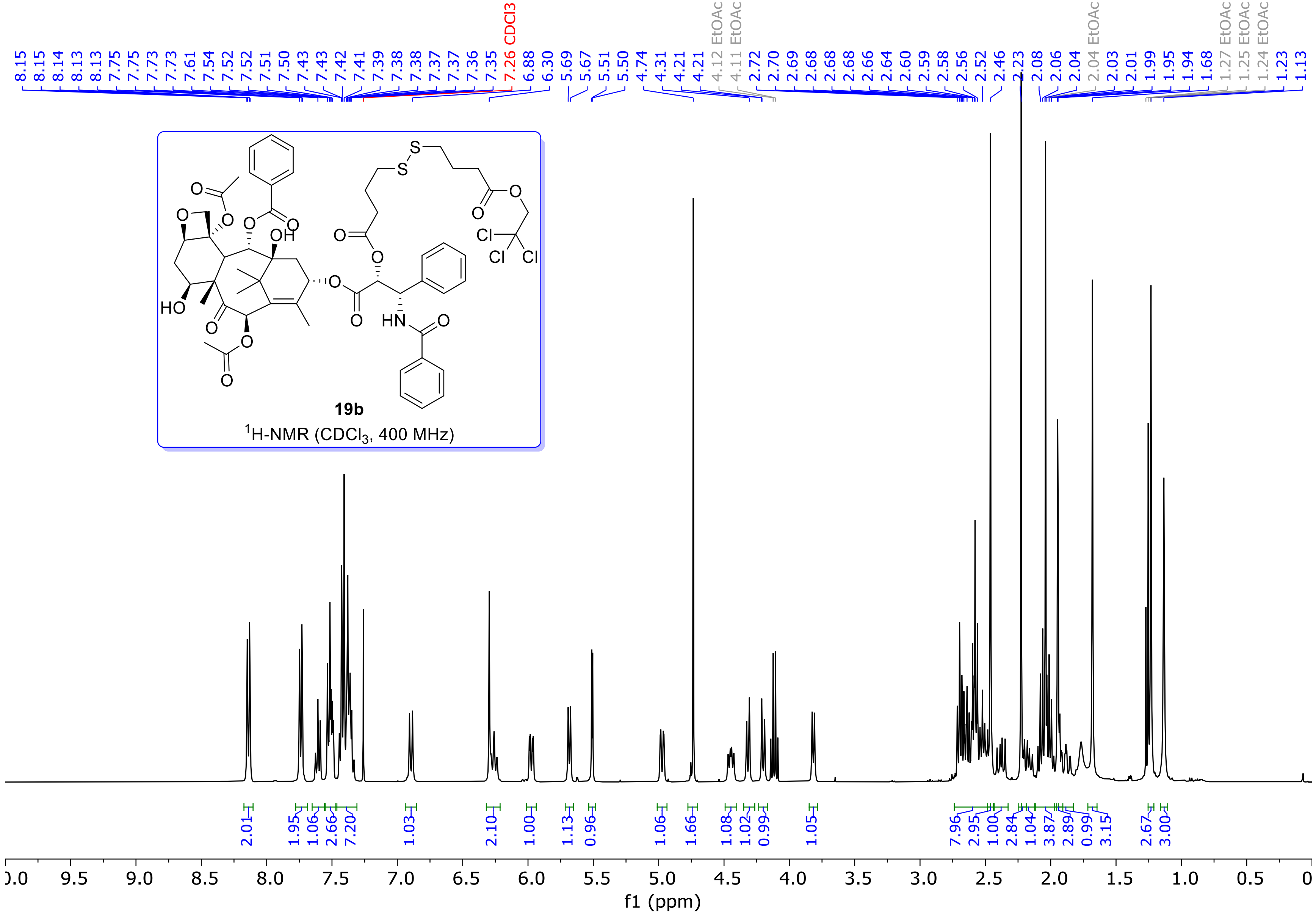
8.15 8.15 8.14 8.13 8.13 7.75 7.75 7.73 7.73 7.61 7.54 7.52 7.52 7.51 7.50 7.43 7.43 7.42 7.41 7.39 7.38 7.38 7.37 7.37 7.36 7.35 7.26 CDCl3 6.88 6.30 5.69 5.67 5.51 5.50 4.74 4.31 4.21 4.21 4.12 EtOAc 4.11 EtOAc 2.72 2.70 2.69 2.68 2.68 2.68 2.66 2.64 2.60 2.59 2.58 2.56 2.52 2.46 2.23 2.08 2.06 2.04 2.04 EtOAc 2.03 2.01 1.99 1.95 1.94 1.68 1.27 EtOAc 1.25 EtOAc 1.24 EtOAc 1.23 1.13
O
O
O
O
OH
O
HO
O
O
O
O
S
S
O
O
O
O
Cl
Cl
Cl
O
HN
O
19b
1H-NMR (CDCl3, 400 MHz)
2.01 1.95 1.06 2.66 7.20 1.03 2.10 1.00 1.13 0.96 1.06 1.66 1.08 1.02 0.99 1.05 7.96 2.95 1.00 2.84 1.04 3.87 2.89 0.99 3.15 2.67 3.00
10.0 9.5 9.0 8.5 8.0 7.5 7.0 6.5 6.0 5.5 5.0 4.5 4.0 3.5 3.0 2.5 2.0 1.5 1.0 0.5 0
f1 (ppm)

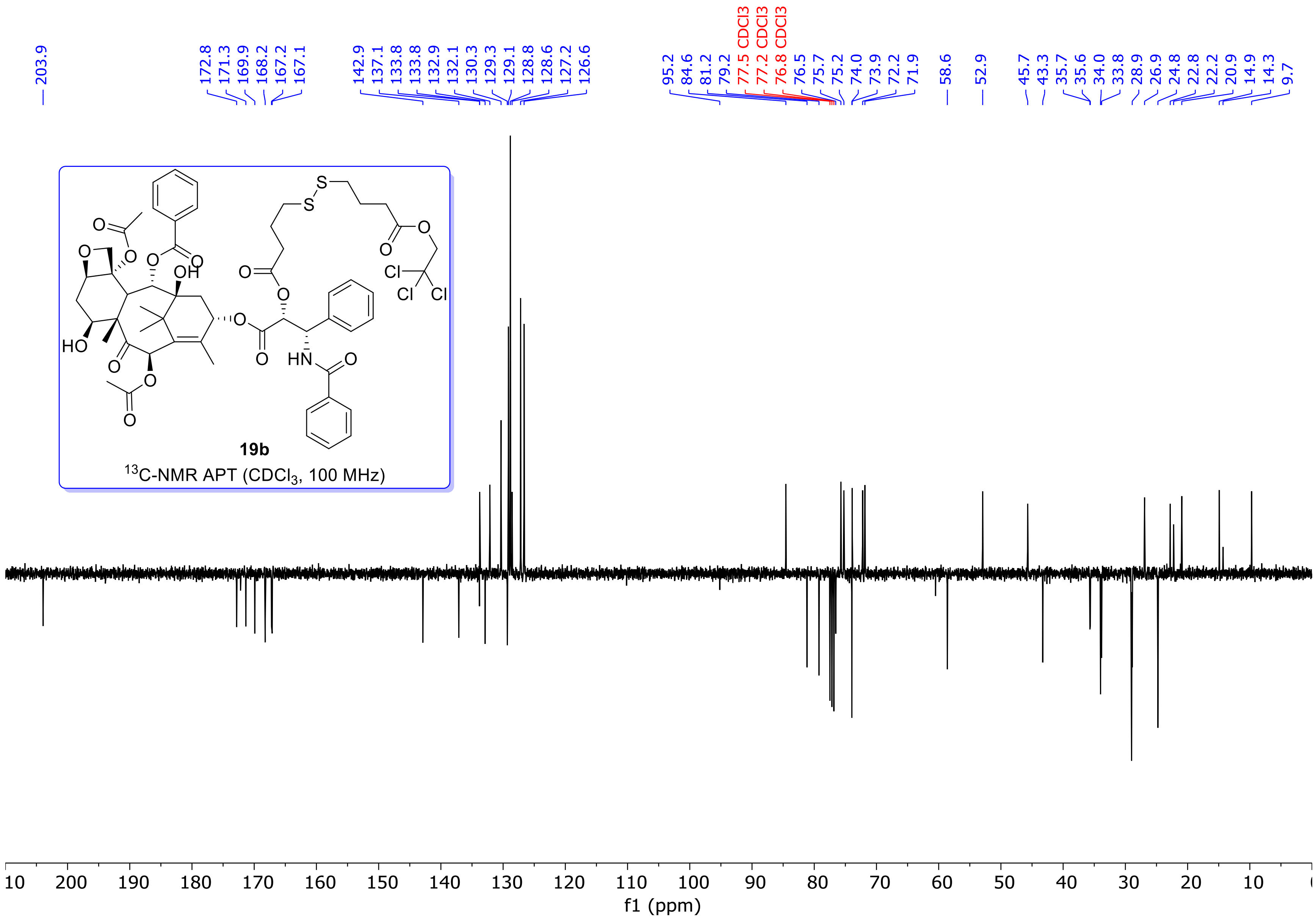
203.9
172.8 171.3 169.9 168.2 167.2 167.1
142.9 137.1 133.8 133.8 132.9 132.1 130.3 129.3 129.1 128.8 128.6 127.2 126.6
95.2 84.6 81.2 79.2 77.5 CDCl3 77.2 CDCl3 76.8 CDCl3 76.5 75.7 75.2 74.0 73.9 72.2 71.9
58.6 52.9
45.7 43.3 35.7 35.6 34.0 33.8 28.9 26.9 24.8 22.8 22.2 20.9 14.9 14.3 9.7
19b
^{13}C-NMR APT ($CDCl_3$, 100 MHz)
f1 (ppm)

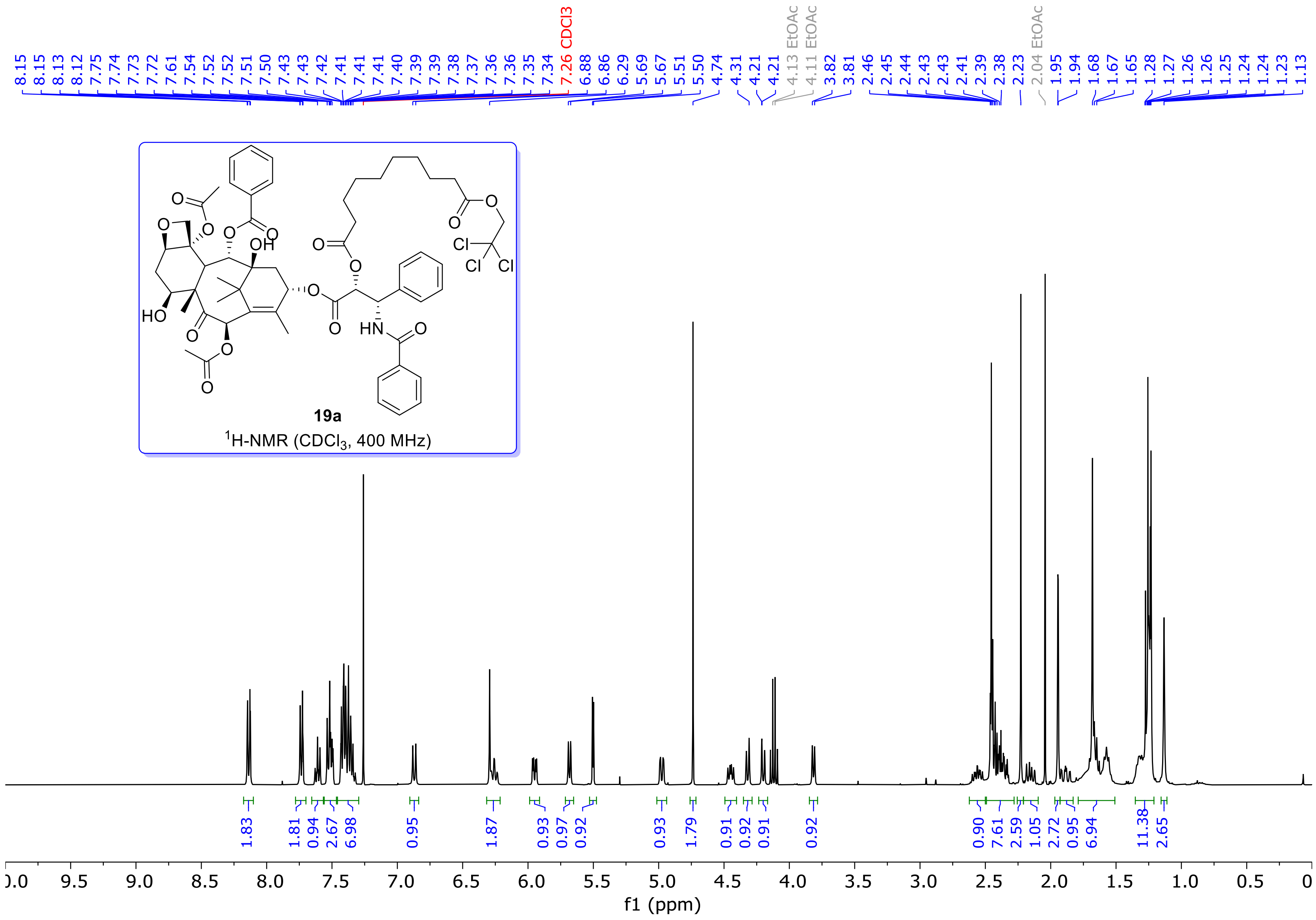
19a
1H-NMR (CDCl3, 400 MHz)
f1 (ppm)

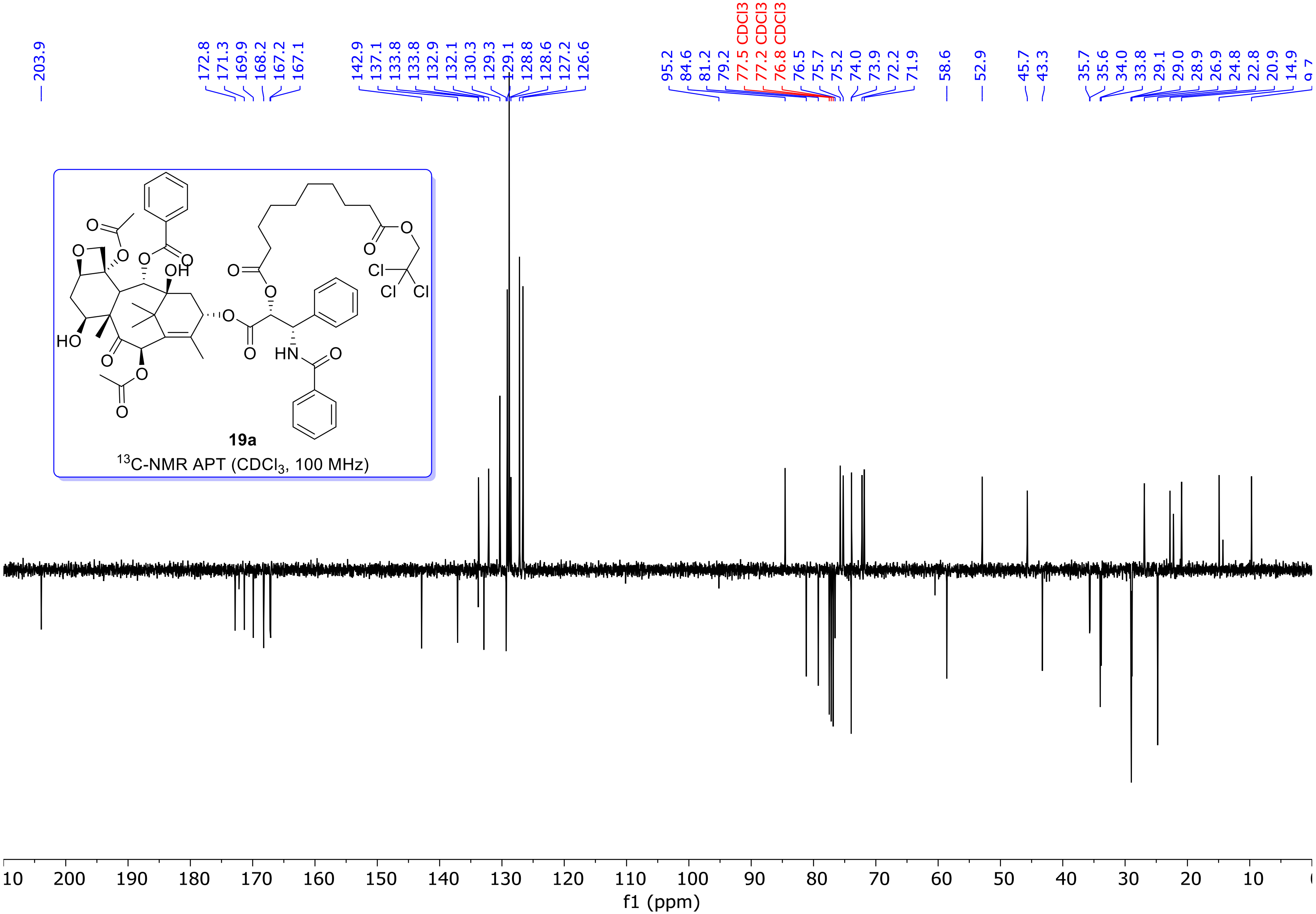

203.9
172.8
171.3
169.9
168.2
167.2
167.1
142.9
137.1
133.8
133.8
132.9
132.1
130.3
129.3
129.1
128.8
128.6
127.2
126.6
95.2
84.6
81.2
79.2
77.5 CDCl3
77.2 CDCl3
76.8 CDCl3
76.5
75.7
75.2
74.0
73.9
72.2
71.9
58.6
52.9
45.7
43.3
35.7
35.6
34.0
33.8
29.1
29.0
28.9
26.9
24.8
22.8
20.9
14.9
9.7
19a
13C-NMR APT (CDCl3, 100 MHz)
200
190
180
170
160
150
140
130
120
110
100
90
80
70
60
50
40
30
20
10
f1 (ppm)

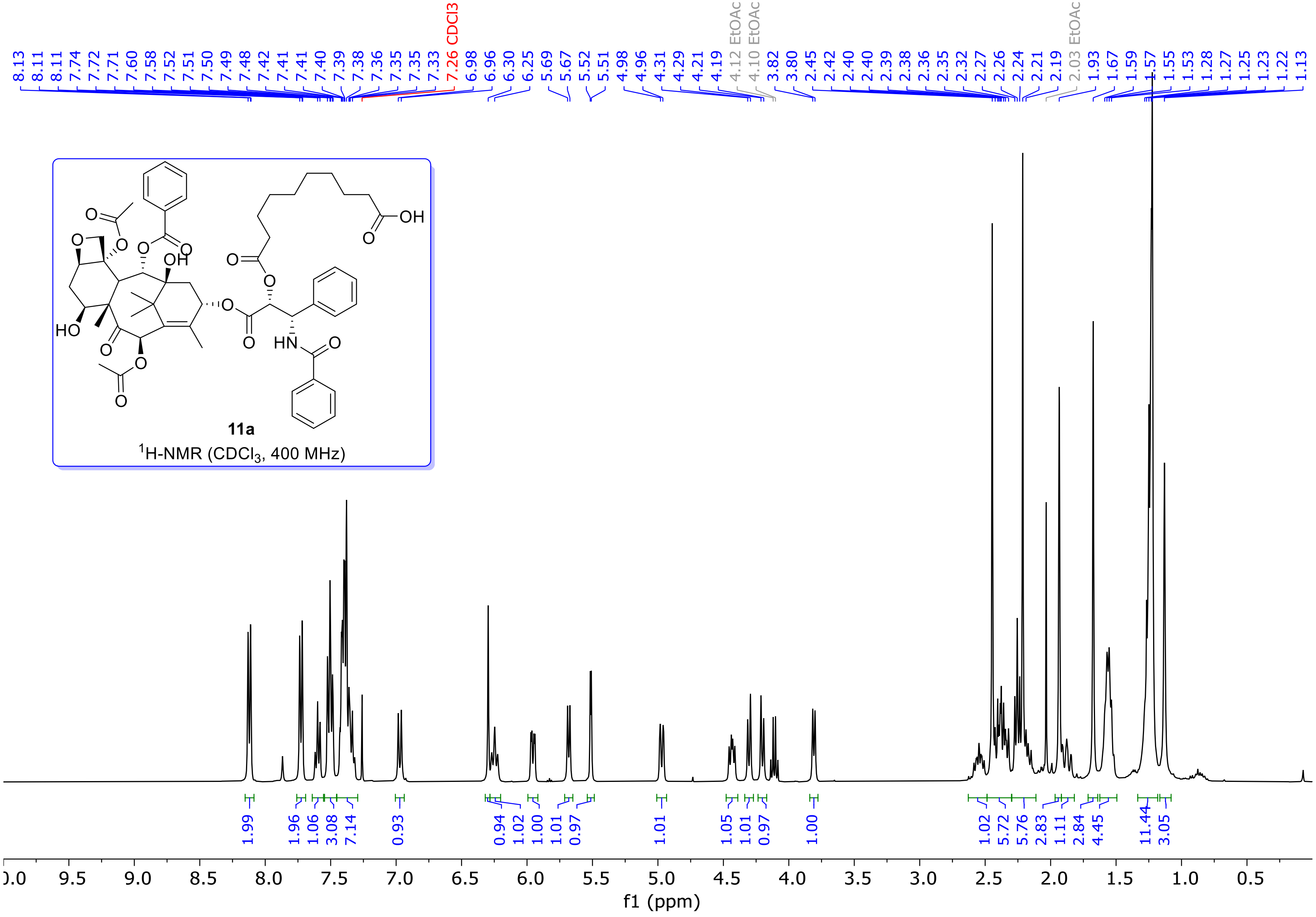
11a
1H-NMR (CDCl3, 400 MHz)
8.13
8.11
8.11
7.74
7.72
7.71
7.60
7.58
7.52
7.51
7.50
7.49
7.48
7.42
7.41
7.40
7.39
7.38
7.36
7.35
7.35
7.33
7.26 CDCl3
6.98
6.96
6.30
6.25
5.69
5.67
5.52
5.51
4.98
4.96
4.31
4.29
4.21
4.19
4.12 EtOAc
4.10 EtOAc
3.82
3.80
2.45
2.42
2.40
2.40
2.39
2.38
2.36
2.35
2.32
2.27
2.26
2.24
2.21
2.19
2.03 EtOAc
1.93
1.67
1.59
1.57
1.55
1.53
1.28
1.27
1.25
1.23
1.22
1.13
1.99
1.96
1.06
3.08
7.14
0.93
0.94
1.02
1.00
1.01
0.97
1.01
1.05
1.01
0.97
1.00
1.02
5.72
5.76
2.83
1.11
2.84
4.45
11.44
3.05
f1 (ppm)

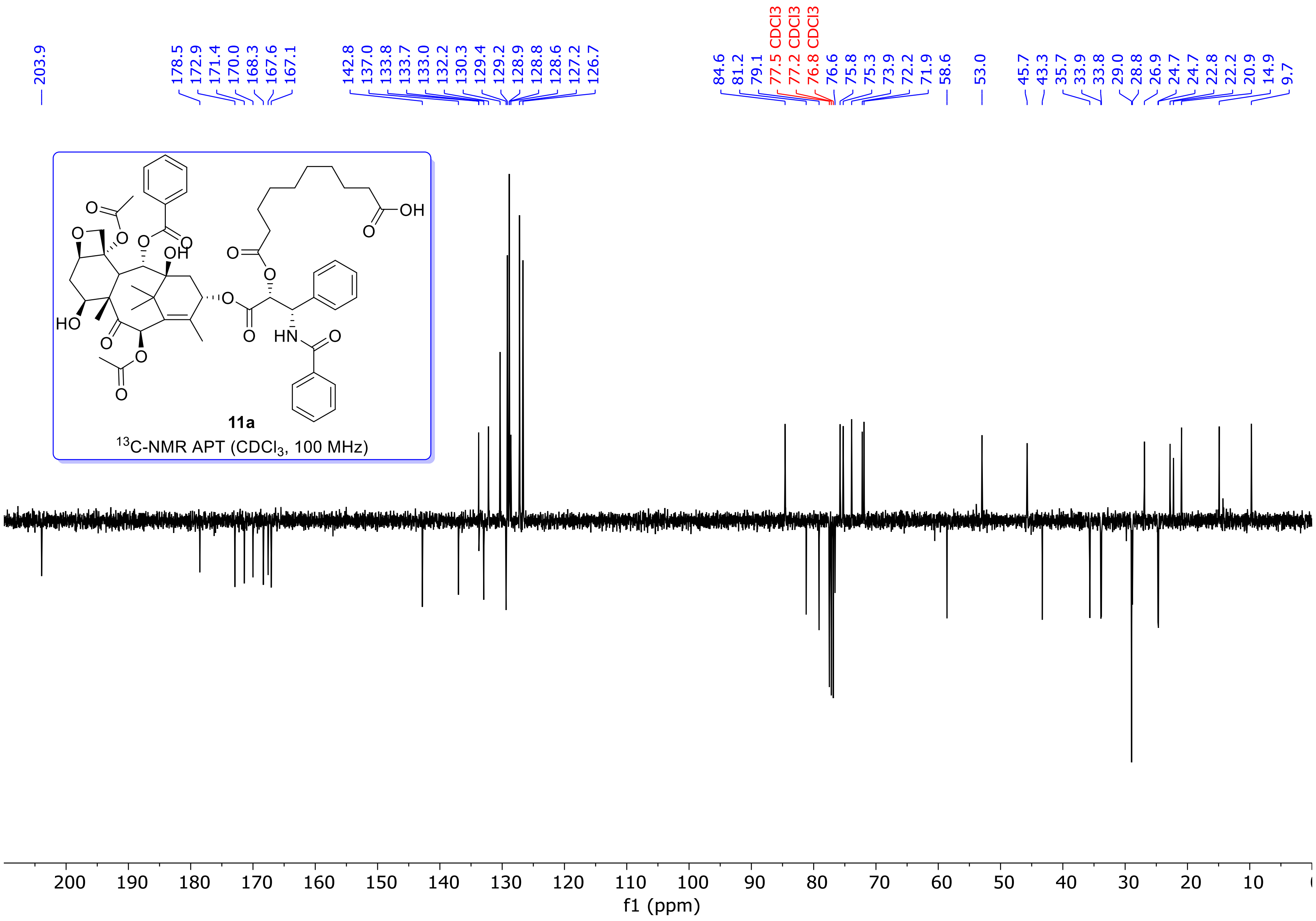
203.9
178.5
172.9
171.4
170.0
168.3
167.6
167.1
142.8
137.0
133.8
133.7
133.0
132.2
130.3
129.4
129.2
128.9
128.8
128.6
127.2
126.7
84.6
81.2
79.1
77.5 CDCl3
77.2 CDCl3
76.8 CDCl3
76.6
75.8
75.3
73.9
72.2
71.9
58.6
53.0
45.7
43.3
35.7
33.9
33.8
29.0
28.8
26.9
24.7
24.7
22.8
22.2
20.9
14.9
9.7
11a
13C-NMR APT (CDCl3, 100 MHz)
200
190
180
170
160
150
140
130
120
110
100
90
80
70
60
50
40
30
20
10
f1 (ppm)

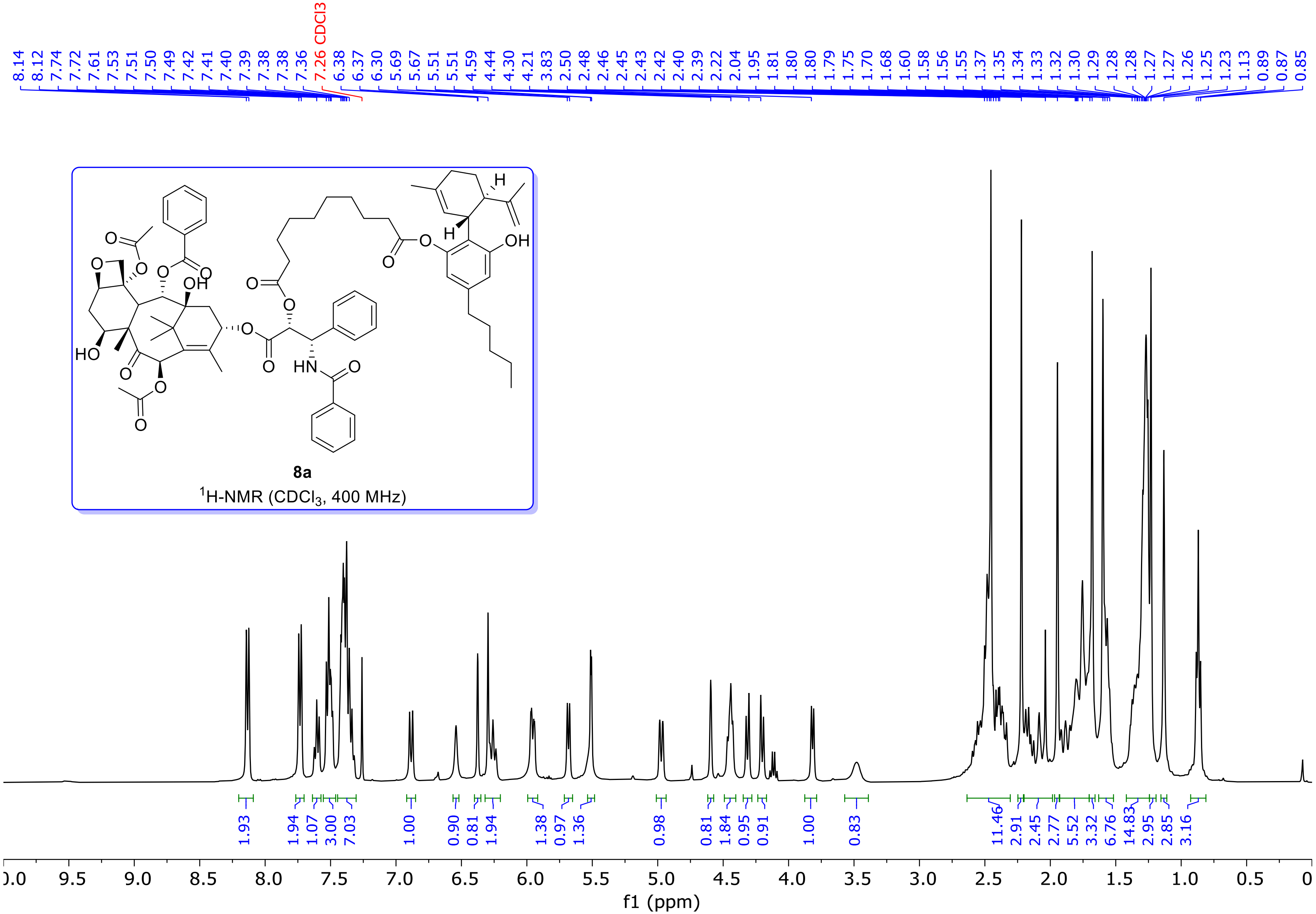

8a

^{1}H-NMR ($CDCl_3$, 400 MHz)

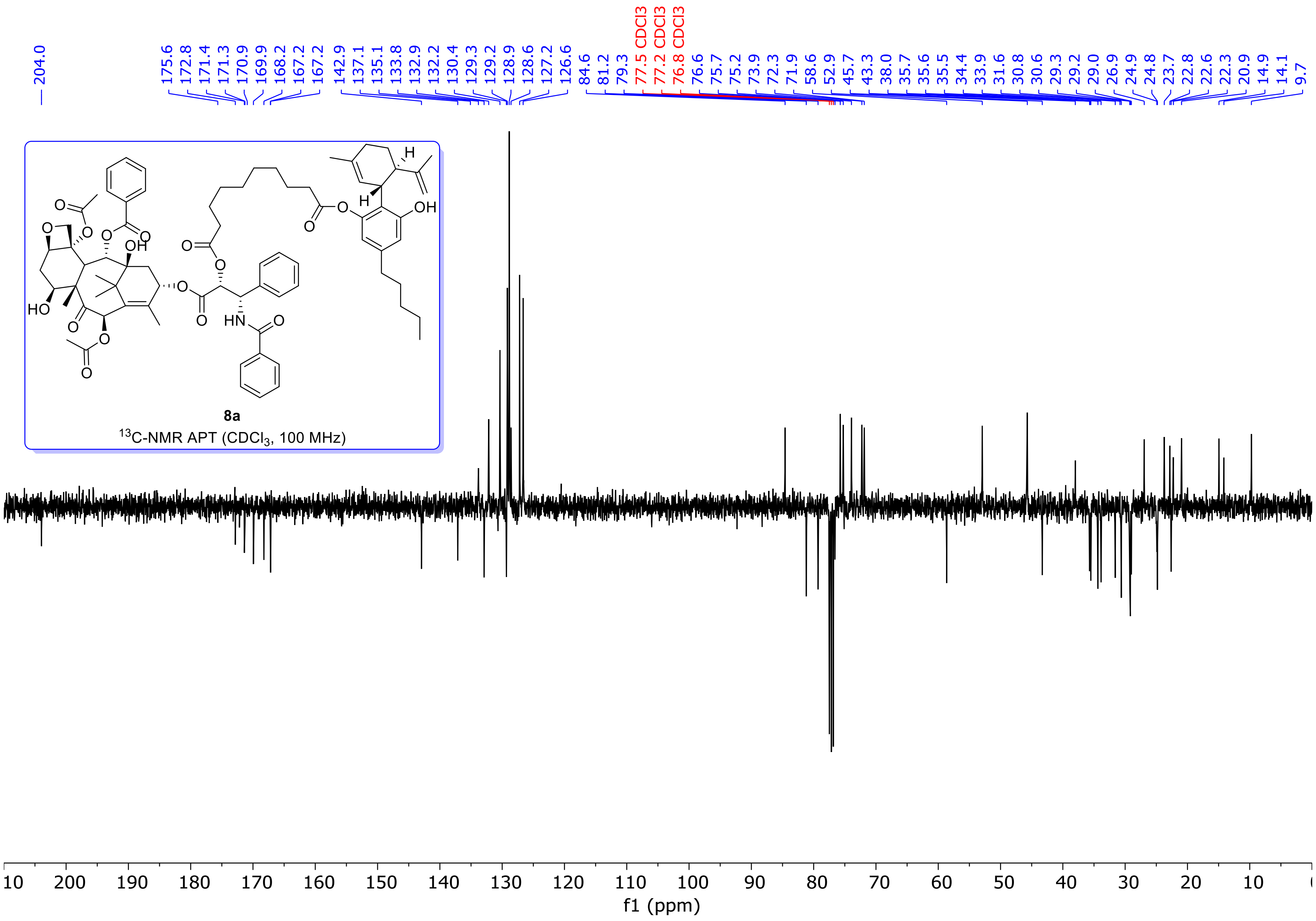
204.0
175.6 172.8 171.4 171.3 170.9 169.9 168.2 167.2 167.2
142.9 137.1 135.1 133.8 132.9 132.2 130.4 129.3 129.2 128.9 128.6 127.2 126.6
84.6 81.2 79.3 77.5 CDCl3 77.2 CDCl3 76.8 CDCl3 76.6 75.7 75.2 73.9 72.3 71.9 58.6 52.9 45.7 43.3 38.0 35.7 35.6 35.5 34.4 33.9 31.6 30.8 30.6 29.3 29.2 29.0 26.9 24.9 24.8 23.7 22.8 22.6 22.3 20.9 14.9 14.1 9.7
O
OH
HO
HN
H
8a
^{13}C-NMR APT (CDCl$_3$, 100 MHz)
f1 (ppm)

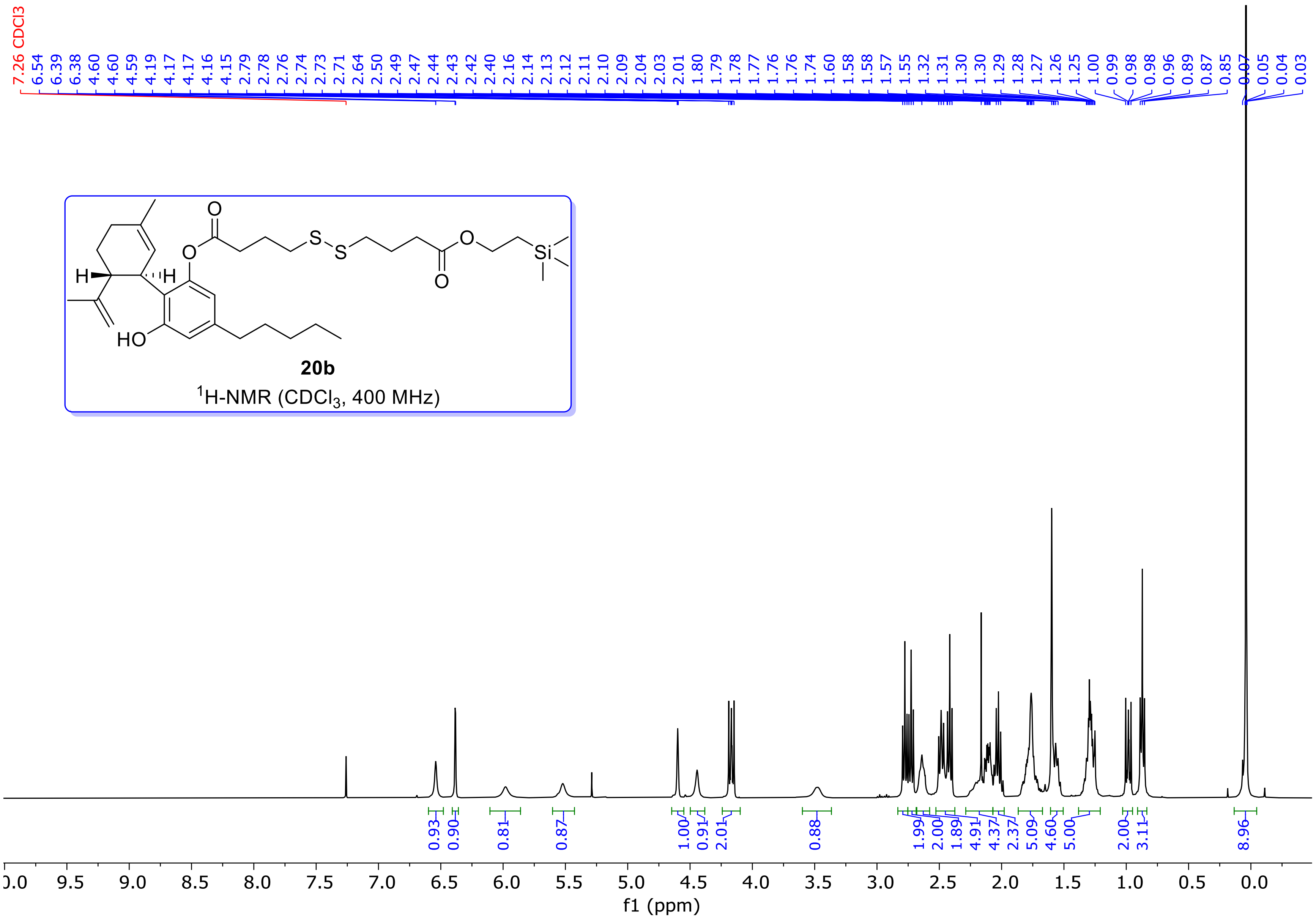
7.26 CDCl3
6.54 6.39 6.38 4.60 4.60 4.59 4.19 4.17 4.17 4.16 4.15 2.79 2.78 2.76 2.74 2.73 2.71 2.64 2.50 2.49 2.47 2.44 2.43 2.42 2.40 2.16 2.14 2.13 2.12 2.11 2.10 2.09 2.04 2.03 2.01 1.80 1.79 1.78 1.77 1.76 1.76 1.74 1.60 1.58 1.58 1.57 1.55 1.32 1.31 1.30 1.30 1.29 1.28 1.27 1.26 1.25 1.00 0.99 0.98 0.98 0.96 0.89 0.87 0.85 0.07 0.05 0.04 0.03
20b
1H-NMR (CDCl3, 400 MHz)
0.93 0.90 0.81 0.87 1.00 0.91 2.01 0.88 1.99 2.00 1.89 4.91 4.37 2.37 5.09 4.60 5.00 2.00 3.11 8.96
f1 (ppm)

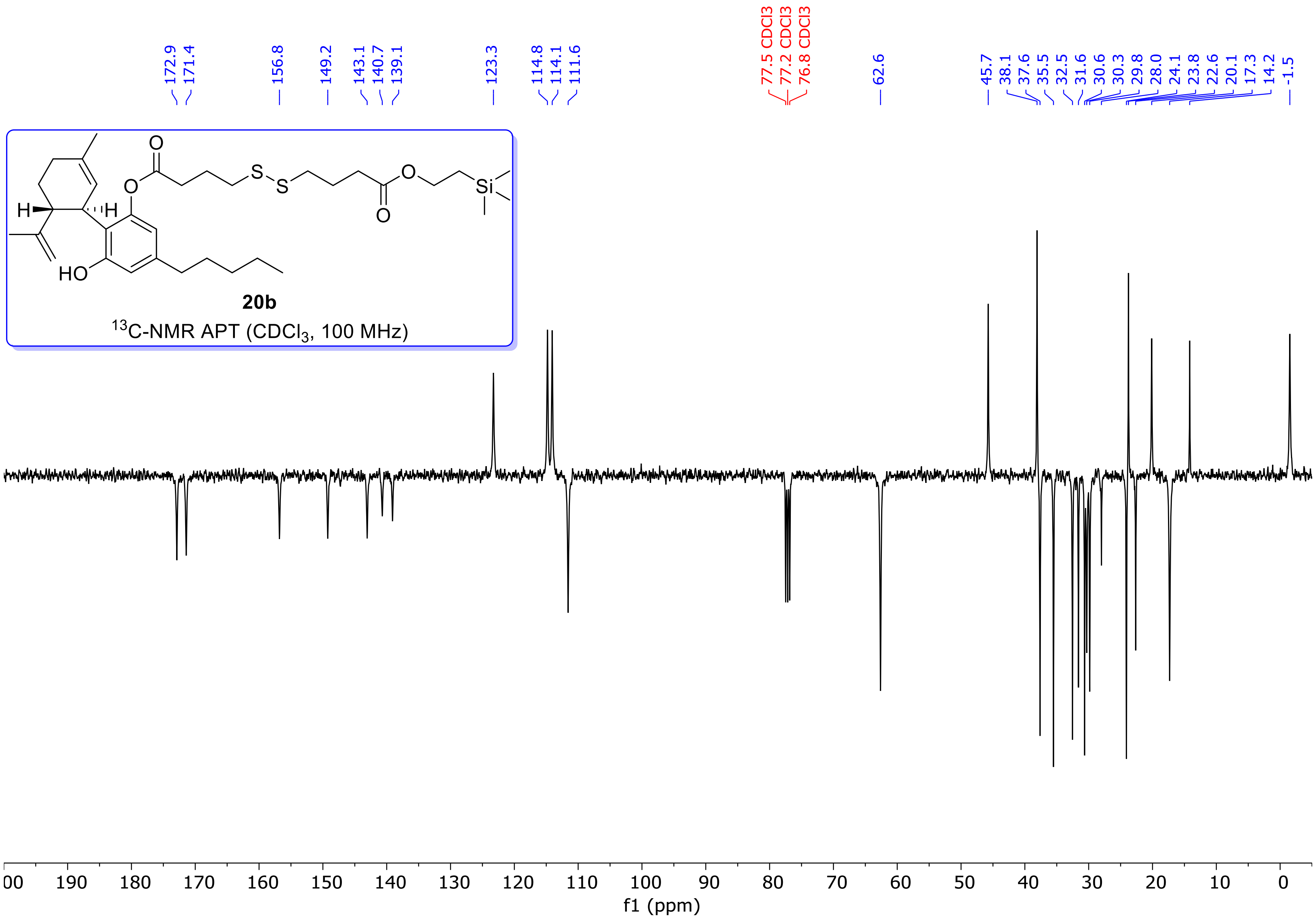
172.9
171.4
156.8
149.2
143.1
140.7
139.1
123.3
114.8
114.1
111.6
77.5 CDCl3
77.2 CDCl3
76.8 CDCl3
62.6
45.7
38.1
37.6
35.5
32.5
31.6
30.6
30.3
29.8
28.0
24.1
23.8
22.6
20.1
17.3
14.2
-1.5
O
S
S
O
O
Si
H
H
HO
20b
13C-NMR APT (CDCl3, 100 MHz)
190
180
170
160
150
140
130
120
110
100
90
80
70
60
50
40
30
20
10
0
f1 (ppm)

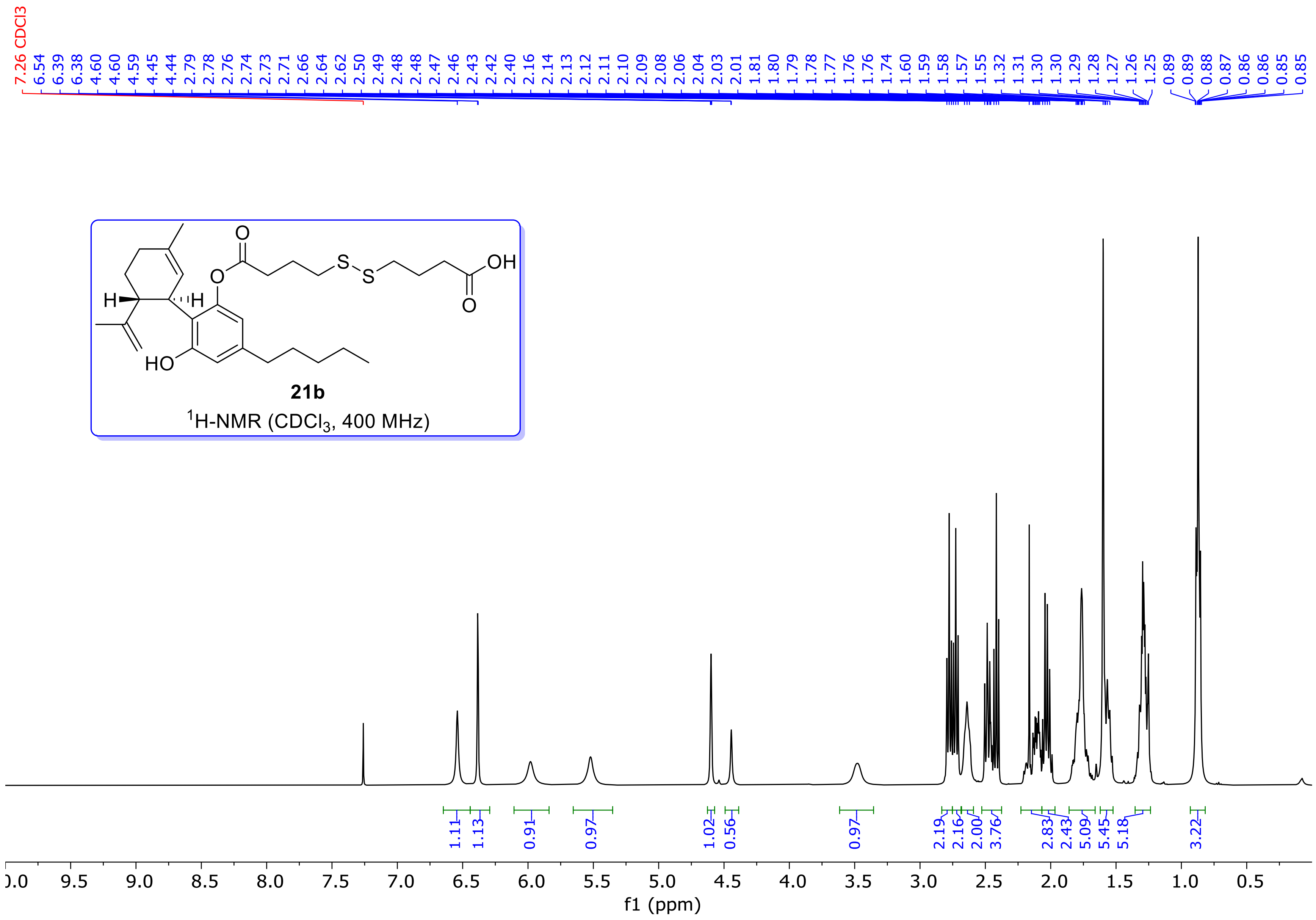
7.26 CDCl3
6.54 6.39 6.38 4.60 4.60 4.59 4.45 4.44 2.79 2.78 2.76 2.74 2.73 2.71 2.66 2.64 2.62 2.50 2.49 2.48 2.48 2.47 2.46 2.43 2.42 2.40 2.16 2.14 2.13 2.12 2.11 2.10 2.09 2.08 2.06 2.04 2.03 2.01 1.81 1.80 1.79 1.78 1.77 1.76 1.76 1.74 1.60 1.59 1.58 1.57 1.55 1.32 1.31 1.30 1.30 1.29 1.28 1.27 1.26 1.25 0.89 0.89 0.88 0.87 0.86 0.86 0.85 0.85
O
OH
S
S
O
H
H
HO
21b
¹H-NMR (CDCl₃, 400 MHz)
1.11 1.13 0.91 0.97 1.02 0.56 0.97 2.19 2.16 2.00 3.76 2.83 2.43 5.09 5.45 5.18 3.22
10.0 9.5 9.0 8.5 8.0 7.5 7.0 6.5 6.0 5.5 5.0 4.5 4.0 3.5 3.0 2.5 2.0 1.5 1.0 0.5
f1 (ppm)

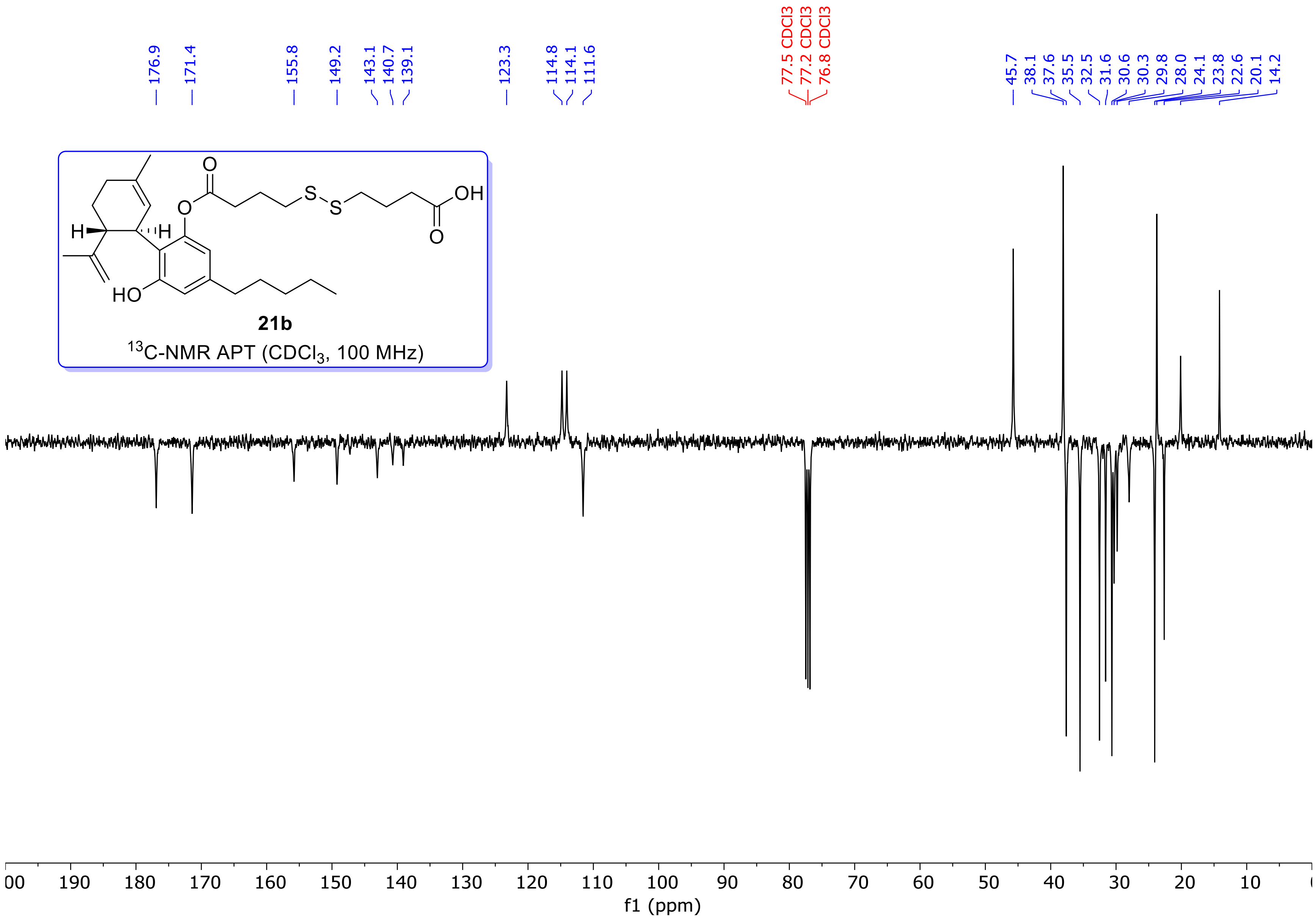
176.9
171.4
155.8
149.2
143.1
140.7
139.1
123.3
114.8
114.1
111.6
77.5 CDCl3
77.2 CDCl3
76.8 CDCl3
45.7
38.1
37.6
35.5
32.5
31.6
30.6
30.3
29.8
28.0
24.1
23.8
22.6
20.1
14.2
21b
13C-NMR APT (CDCl3, 100 MHz)
f1 (ppm)

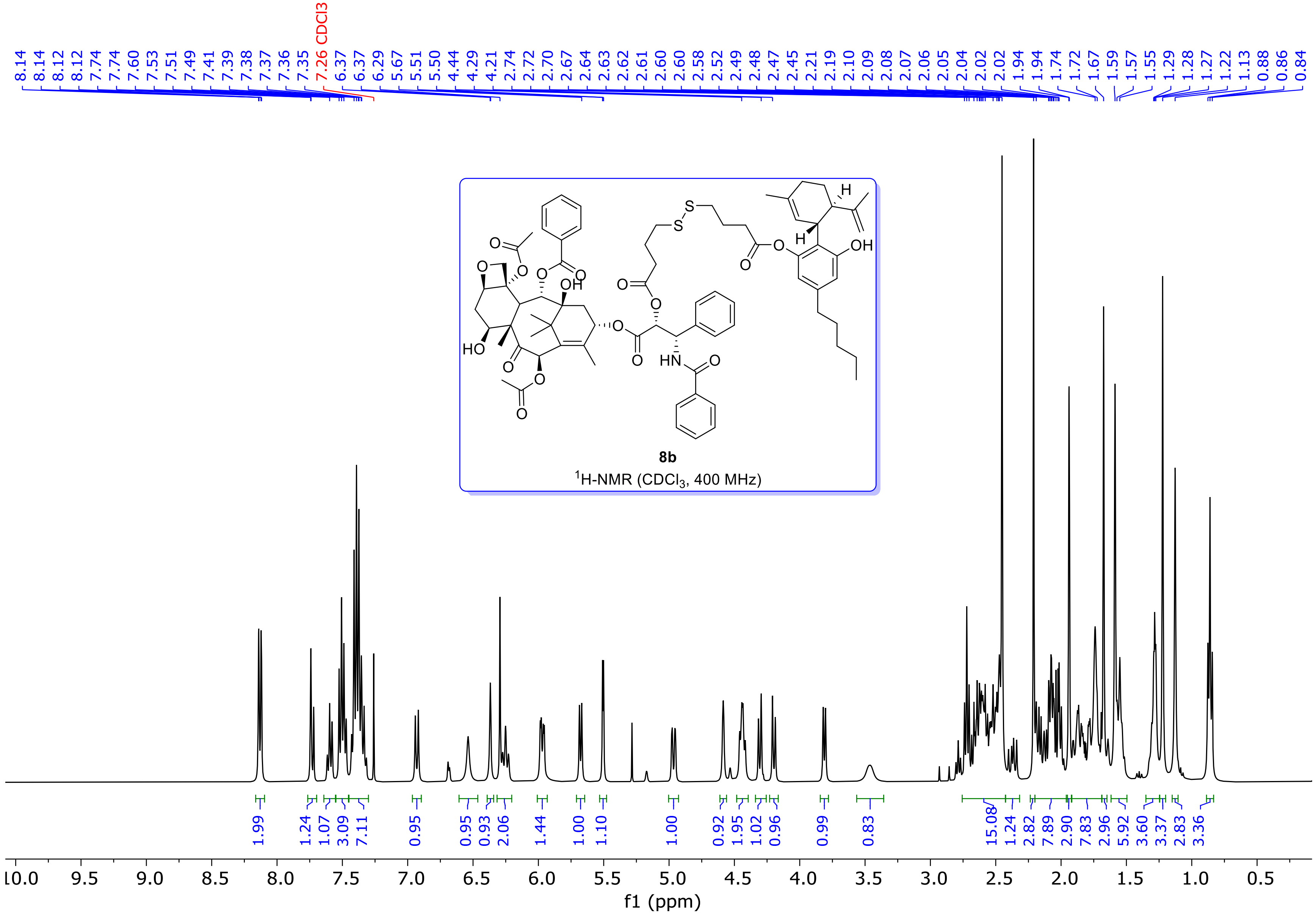

8b
^{1}H-NMR ($CDCl_3$, 400 MHz)
f1 (ppm)

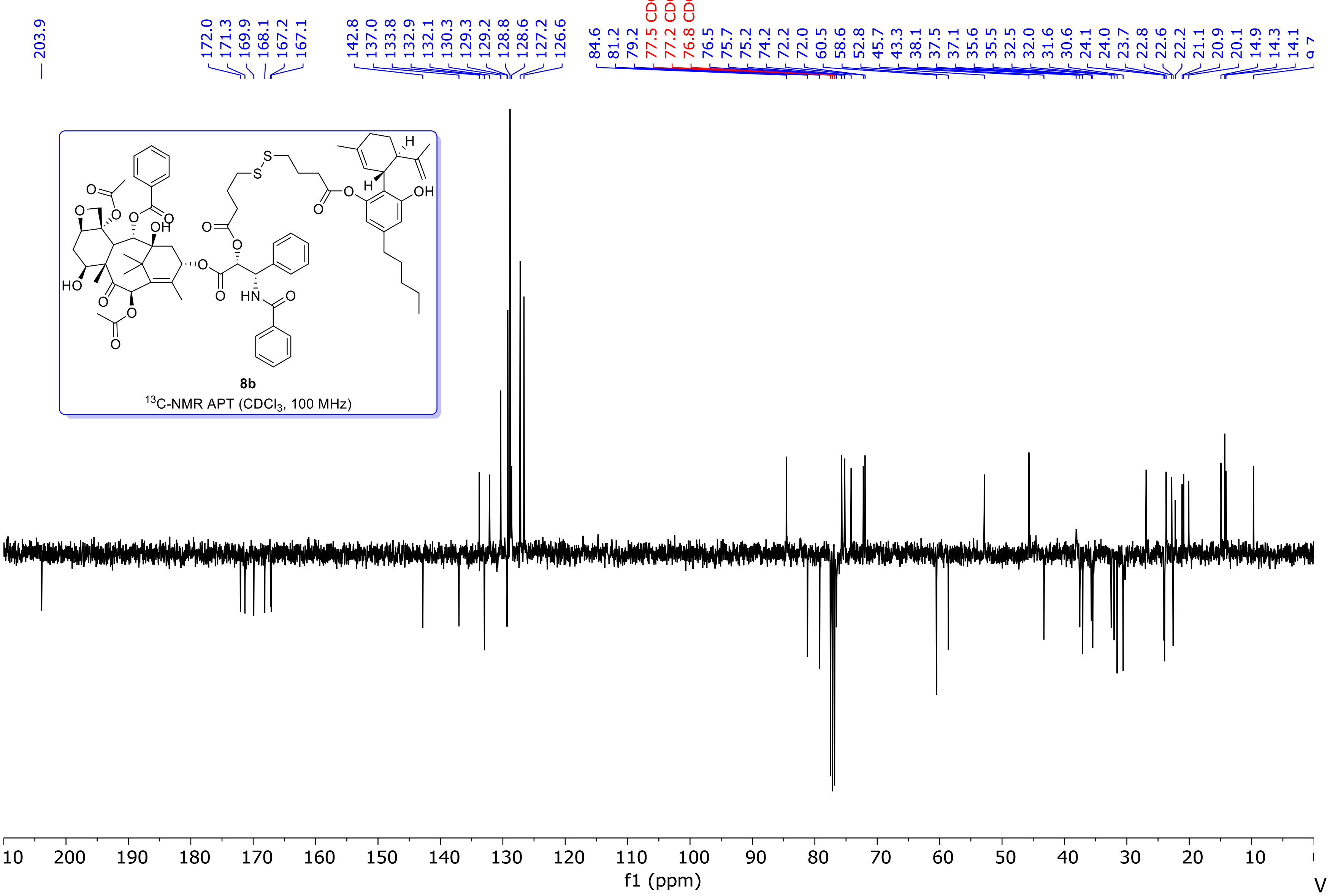

203.9
172.0
171.3
169.9
168.1
167.2
167.1
142.8
137.0
133.8
132.9
132.1
130.3
129.3
129.2
128.8
128.6
127.2
126.6
84.6
81.2
79.2
77.5 CDCl3
77.2 CDCl3
76.8 CDCl3
76.5
75.7
75.2
74.2
72.2
72.0
60.5
58.6
52.8
45.7
43.3
38.1
37.5
37.1
35.6
35.5
32.5
32.0
31.6
30.6
24.1
24.0
23.7
22.8
22.6
22.2
21.1
20.9
20.1
14.9
14.3
14.1
8b
13C-NMR APT (CDCl3, 100 MHz)
210
200
190
180
170
160
150
140
130
120
110
100
90
80
70
60
50
40
30
20
10
f1 (ppm)
